# MS-MDDNet: A Lightweight Deep Learning Framework for Interpretable EEG-Based Diagnosis of Major Depressive Disorder

**DOI:** 10.3390/diagnostics16020363

**Published:** 2026-01-22

**Authors:** Rabeah AlAqel, Muhammad Hussain, Saad Al-Ahmadi

**Affiliations:** Department of Computer Science, College of Computer and Information Sciences, King Saud University, Riyadh 11451, Saudi Arabiasalahmadi@ksu.edu.sa (S.A.-A.)

**Keywords:** deep learning, convolutional neural networks (CNN), interpretability, Major Depressive Disorder (MDD), electroencephalography (EEG)

## Abstract

**Background**: Major Depressive Disorder (MDD) is a pervasive psychiatric condition. Electroencephalography (EEG) is employed to detect MDD-specific neural patterns because it is non-invasive and temporally precise. However, manual interpretation of EEG signals is labor-intensive and subjective. This problem was addressed by proposing machine learning (ML) and deep learning (DL) methods. Although DL methods are promising for MDD detection, they face limitations, including high model complexity, overfitting due to subject-specific noise, excessive channel requirements, and limited interpretability. **Methods**: To address these challenges, we propose MS-MDDNet, a new lightweight CNN model specifically designed for EEG-based MDD detection, along with an ensemble-like method built on it. The architecture of MS-MDDNet incorporates spatial, temporal, and depth-wise separable convolutions, along with average pooling, to enhance discriminative feature extraction while maintaining computational efficiency with a small number of learnable parameters. **Results:** The method was evaluated using 10-fold Cross-Subjects Cross-Validation (CS-CV), which mitigates the risks of overfitting associated with subject-specific noise, thereby contributing to generalization robustness. Across three public datasets, the proposed method achieved performance comparable to state-of-the-art approaches while maintaining lower computational complexity. It achieved a 9% improvement on the MODMA dataset, with an accuracy of 99.33%, whereas on MUMTAZ and PRED + CT it achieved accuracies of 98.59% and 96.61%, respectively. **Conclusions**: The predictions of the proposed method are interpretable, with interpretability achieved through correlation analysis between gamma energy and learned features. This makes it a valuable tool for assisting clinicians and individuals in diagnosing MDD with confidence, thereby enhancing transparency in decision-making and promoting clinical credibility.

## 1. Introduction

Major Depressive Disorder (MDD) is increasingly recognized as a widespread and debilitating psychiatric disorder that profoundly affects individuals’ emotional, cognitive, and physiological functioning. EEG signals potentially serve as depression biomarkers and reflect complex brain properties relevant to MDD [1]. Manual analysis of EEG signals is labor-intensive, time-consuming, and prone to inter-observer variability, underscoring the need for automated machine learning solutions [2,3,4,5,6,7,8,9,10,11,12,13,14,15,16,17,18,19,20,21,22,23,24,25]. Traditional machine learning (ML) methods typically depend on manually engineered features, a process that is both resource-intensive and prone to suboptimal outcomes.

The limitations of traditional ML methods have led to the growing adoption of deep learning techniques. Various deep learning-based architectures, such as convolutional neural networks (CNNs) [13], long-short-term memory (LSTM) [26], graph neural networks (GNNs) [27], and transformers [28], have been adopted for MDD detection. However, CNNs and their variants, such as 1D-CNN [10,29,30], 2D-CNN [31,32,33,34], and 3D-CNN [35,36], have been increasingly used for MDD detection due to their ability to learn rich representations directly from EEG signals. Despite their strong performance, CNN models, particularly 2D-CNNs and 3D-CNNs, are highly susceptible to overfitting, largely due to subject-specific noise inherent in EEG data [37]. Although recent studies have introduced architectures designed to mitigate this issue [27,28,29,33,34,38,39,40], these approaches face notable constraints. Specifically, highly complex deep architectures contribute to elevated computational cost and memory usage. Additionally, many models rely on a large number of EEG channels as input, posing practical challenges for integrating them into user-friendly and portable systems. Most critically, the limited interpretability of these models remains a significant barrier to clinical adoption, hindering transparency and scalability in healthcare applications.

The evaluation procedure plays a critical role in model design and reliability assessment. SI-CV protocols often yield inflated performance due to potential overlap between training and test subjects, as evidenced by consistently higher accuracy reported in [12,16,17]. In contrast, the CS-CV protocol provides a more realistic measure of generalization. Studies adopting CS-CV with deep learning [28,29,33] are comparatively fewer and face issues related to interpretability and deployment.

To address the limitations inherent in the state-of-the-art approaches, we propose MS-MDDNet, a lightweight neural network model and an efficient ensemble-like classification framework based on it. MS-MDDNet is specifically designed to capture both spatial and temporal characteristics embedded within EEG signals pertinent to Major Depressive Disorder (MDD). It integrates spatial-temporal convolutions, depth-wise separable convolutions, and average pooling to extract salient features while maintaining computational efficiency and reducing the risk of overfitting. MS-MDDNet is a compact neural network model characterized by a small number of trainable parameters. Unlike conventional ensemble learning methods [18], which typically involve multiple base learners, our ensemble-like classification framework relies on a single model. It introduces diversity by segmenting the input EEG signals rather than relying on multiple models. To assess its generalizability and robustness against subject-specific noise, the model has been rigorously evaluated using 10-fold CS-CV across three publicly available benchmark datasets. Furthermore, an intrinsic interpretability mechanism has been introduced by analyzing the correlation between the model’s learned features and the gamma band, thereby enhancing transparency in decision-making and promoting clinical credibility.

The novelty of this study lies in the design and implementation of MS-MDDNet, a compact, end-to-end deep learning architecture specifically optimized for EEG-based MDD detection. The main contributions are as follows:

- **MS-MDDNet, a novel lightweight CNN model**: Unlike conventional CNN or hybrid models, MS-MDDNet integrates *multi-scale spatial-temporal convolutions* with *depthwise separable convolutions* and *average pooling* in a compact design. This architecture is specifically designed for EEG signals to enhance discriminative feature extraction while reducing computational complexity and the risk of overfitting. This architecture has not been used in existing MDD detection methods.
- **Ensemble-like Classification Framework**: It applies the ensemble principle by dividing longer inference-time EEG trials (e.g., 10 s) into sub-trials (e.g., 2 s), which naturally capture diverse temporal patterns due to the non-stationary and noisy nature of EEG signals [18]. Each sub-trial is treated as an independent observation, and aggregating the predictions across all sub-trials yields a more robust and reliable final decision. This approach captures diverse temporal patterns, mitigates noise, and improves stability, leading to better generalization and higher accuracy compared to single-segment predictions. Additionally, it allows MS-MDDNet to be designed as a lightweight model trained on a large number of short trials (e.g., 2 s), reducing the risk of overfitting with limited data. This strategy avoids the complexity and storage requirements of conventional ensembles, making it an efficient *ensemble-like classification method*.
- **Channel Efficiency**: Existing deep learning approaches often require a large number of EEG channels, limiting scalability. Our model achieves high accuracy with minimal channels, reducing hardware dependency and setup complexity, which is essential for real-world deployment.
- **An Intrinsic Interpretability Mechanism**: To enhance transparency and clinical credibility, we introduced an interpretability method that is based on the analysis of the correlation between the model’s learned features and the clinically relevant gamma band in EEG signals.
- **A Rigorous Evaluation Strategy**: We demonstrated the model’s generalizability and robustness through extensive evaluation using 10-fold CS-CV across three publicly available benchmark datasets. Our use of 10-fold CS-CV ensures robust generalization and mitigates overfitting due to subject-specific noise, a methodological improvement that strengthens clinical applicability.
- **Superior Performance with Reduced Complexity**: Compared to state-of-the-art methods, MS-MDDNet achieves higher accuracy and F1 scores across multiple datasets while maintaining a lightweight architecture, making it both practical and clinically viable.

The remainder of the paper is organized as follows: Section 2 provides details of the proposed MS-MDDNet architecture and the ensemble framework, while Section 3 describes the evaluation protocol adopted in this study and presents and analyzes the experimental results and the method’s robustness. Section 4 explores the influence of various brain rhythms on MS-MDDNet’s decision-making process, investigates the correlation between the learned features and gamma rhythm, and introduces an interpretable strategy for explaining model predictions. Finally, Section 5 concludes the paper.

## 2. Proposed Method

### 2.1. Problem Formulation

The brain activity of a subject is recorded using electroencephalography (EEG), producing an EEG signal (referred to as a trial or epoch) that reflects neural activations over a specific time window. This EEG trial is then analyzed to determine whether the subject is a healthy control (HC) or exhibits signs of MDD. The objective is to develop an automated method capable of distinguishing between HC and MDD based on EEG epochs. Given the two target classes, HC and MDD, the detection task is formulated as a binary classification problem.

Formally, let an EEG trial be represented as a matrix x $\in R^{m\times n}:$ i.e.,

(1) $$x=\left[\begin{array}{c} \begin{array}{c} x_{11\;}{\;\;\;x}_{12\;}\;\cdots {\;\;x}_{1n\;}\\ x_{21\;}{\;\;\;x}_{22\;}\;\cdots {\;\;x}_{2n\;} \end{array}\\ x_{31\;}{\;\;\;x}_{32\;}\;\cdots {\;\;x}_{3n}\\ \vdots \;\;\;\;\;\;\;\;\vdots \;\;\;\;\;\;\;\vdots \;\;\;\;\;\;\;\vdots \\ x_{m1\;}{\;\;\;x}_{m2\;}\;\cdots {\;\;x}_{mn} \end{array}\right]$$

where m represents the number of channels (spatial dimension) and n= sΤ is the number of time samples (temporal dimension) recorded with the sampling rate s over the time interval of Τ seconds. Each row represents a channel, and each column denotes one time sample recorded from m  electrodes. Let Y = {0,1} represent the set of labels, where a label of 0 corresponds to HC and a label of 1 corresponds to MDD. The problem of detecting MDD is framed as a classification problem, where x is the input and l is the corresponding output, i.e., the predicted label of x. We need to design a mapping f( . ; *θ*): $\in R^{m\times n}$ → Y such that f(x; *θ*) = l, where $x\in R^{m\times n}$ and l ∈ Y. In this mapping, *θ* represents the learnable parameters of f; its complexity depends on the number of learnable parameters. As the deep learning approach enables learning a mapping *f* in an end-to-end manner, we design *f* as a lightweight deep neural network model involving a small number of learnable parameters θ. It ensures avoiding overfitting, as the EEG data for MDD is limited in its capacity to learn a deep model. The details of the architecture of a deep neural network, called MS-MDDNet, representing f is presented in the following subsections.

### 2.2. MS-MDDNet Architecture

**Figure 1:**
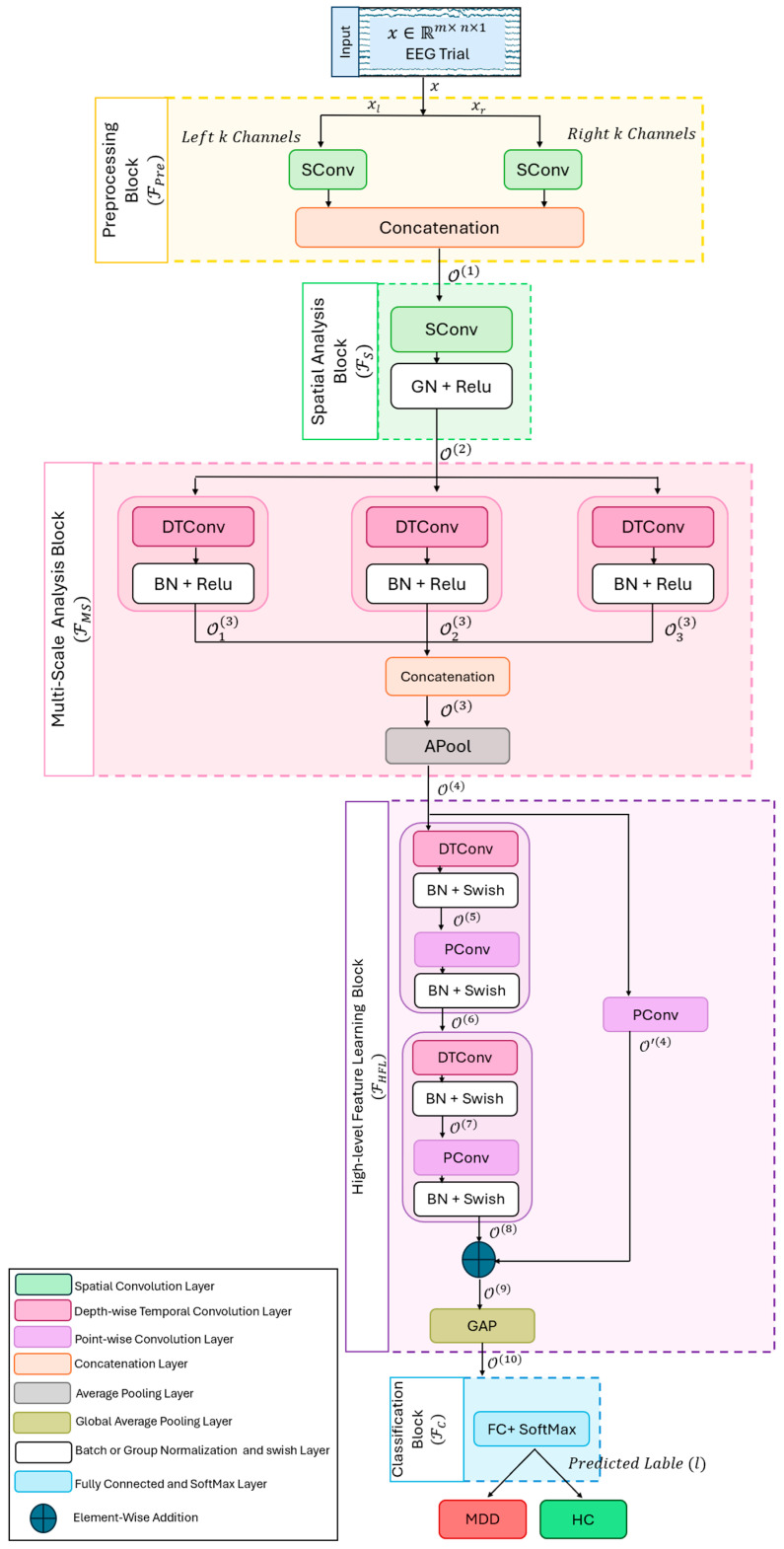
The MS-MDDNet Architecture.

MS-MDDNet is a lightweight model designed to classify an EEG trial *x* and predict whether a subject is affected by MDD or is an HC. The proposed architecture overcomes the limitations of prior methods by enhancing model efficiency, reducing complexity, and improving generalization performance. MS-MDDNet integrates various types of convolution operations, including multiscale, depth-wise, spatial, and separable convolutions, to effectively capture diverse signal patterns. The model is structured into five main mappings: Spatial Information Integration ($F_{Pre}$), Spatial Analysis ($F_{S}$), Multiscale Analysis ($F_{MS}$), High-level Feature Learning ($F_{HFL}$), and the Classifier ($F_{c}$). These mappings collectively define f(.; θ): $\in R^{m\times n}$→ Y, representing MS-MDDNet, as follows:

(2) $$f(x;\;\theta )\;=\;F_{C}\;{\circ \;F}_{HFL}\;{\circ \;F}_{MS}\;\circ \;F_{S}{\;\circ F}_{Pre}\left(x\right),$$

where *θ* = {*θ_Pre_*, *θ_S_*, *θ_MS_*, *θ_HFL_*, *θ_C_*} represents the learnable parameters of $F_{Pre},F_{S},$ $F_{MS},F_{HFL}\;and\;F_{C},$ respectively. The subsequent subsections elaborate on each of these mappings in detail. Figure 1 illustrates the MDDNet framework, with its mappings represented as block diagrams; Table 1 presents the corresponding specifications. Following the definition of an EEG trial in Equation (1), the input size 32 × 500 × 1 represents that the input is an EEG trial that has *m* = 32 channels, and *n* = 500 time samples.

#### 2.2.1. Spatial Information Integration ${(F}_{pre})$

An EEG signal records brain activity using electrodes placed at various locations on the skull. It is essential to integrate this information adaptively to extract patterns relevant to MMD. To this end, this mapping independently fuses channels from both hemispheres based on their spatial location and importance for detecting MDD, as shown in Figure 1. Mathematically, the mapping $F_{Pre}:R^{m\times n\times 1}\to R^{2\times n\times 1}$ is defined as follows:

(3) $$O^{\left(1\right)}=F_{Pre}\left(x;\theta_{Pre}\right)=C\left(O_{r}^{\left(1\right)},O_{l}^{\left(1\right)}\right),$$

where

$$O_{r}^{\left(1\right)}=\psi_{S}^{\left(r\right)}(x_{r};\theta_{s}^{\left(r\right)})$$

$$O_{l}^{\left(1\right)}=\psi_{S}^{\left(l\right)}(x_{l};\theta_{s}^{\left(l\right)})$$

$x=[x_{r};x_{l}$], $x_{r}\in R^{m_{r}\times n\times 1},\;x_{l}\in R^{m_{l}\times n\times 1}$, $m_{r}\;$ and $m_{l}$ are the numbers of channels in $x_{r}$ and $x_{l}$, the parts of x in the right and left hemispheres.

The mapping $\psi_{S}^{\left(r\right)}\;(and\;\psi_{S}^{\left(l\right)})$ is defined as a spatial convolution SConv*_r_* (and SConv*_l_*) layer with one filter of size $m_{r}$ $\times \;1\;\left(and\;m_{l}\times 1\;\right)\;$ and $\theta_{Pre}\;=\;\{\theta_{s}^{\left(r\right)},\theta_{s}^{\left(l\right)}\}$ represent the learnable parameters of the kernels of SConv layers; C is the concatenation.

Applying spatial convolution enables the model to effectively capture the discriminative spatial features across different channel locations and adaptively fuse them. This approach allows integration of discriminative information across different spatial locations by learning patterns specifically indicative of MDD. As a result, the spatial convolutional layer enhances the representation of the input EEG trial, reduces model complexity, and ultimately improves generalization and classification accuracy. The concatenation operation combines the MDD-relevant information from the two hemispheres, making the representation richer and more discriminative.

#### 2.2.2. Spatial Analysis ${(F}_{s})$

The previous mapping adaptively integrates the spatial patterns from each hemisphere. These patterns must be fused to work out the multiple discriminative spatial patterns specific to MDD. This mapping receives $O^{1}$ from $F_{pre}$ and calculates the multiple spatial patterns using spatial convolution with many filters. It consists of a single spatial convolution, followed by feature distribution normalization, and an activation function. Mathematically, it is the mapping ${F_{s}:R}^{2\times n\times 1}\to R^{1\times n\times F_{1}}$ defined as follows:

(4) $$O^{2}=F_{s}\left(x;\;\theta_{sF_{1}}^{\left(3\right)}\right)=G\circ R\circ \psi_{S}^{\left(3\right)}\left(x\right)$$

where $\psi_{S}^{\left(3\right)}$ is spatial convolution with learnable parameters $\theta_{sF_{1}}^{\left(3\right)}$, G is a feature distribution normalization, and R is an activation function. The mapping $\psi_{S}^{\left(3\right)}$ is implemented as a spatial convolutional (SConv) layer with $F_{1}$ filters, and $\theta_{sF_{1}}^{\left(3\right)}$ are learnable parameters of these filters. For feature distribution normalization and activation function, there are different options; empirically, we found that group normalization and ReLU activation give the best performance. The mapping $F_{s}$ produces $O^{2}$ $\in R^{1\times n\times F_{1}}$, which consists of $F_{1}$ temporal channels, each of dimension 1×n.

Spatial convolution is designed to extract distinctive spatial features while maintaining the temporal dimension. It reduces the spatial dimension from two spatial channels to one channel by applying $F_{1}$ spatial filters of size [2 × 1], generating $F_{1}$ temporal feature channels that integrate spatial information from the left and right hemispheres. Unlike batch normalization, grouped normalization (GN) is independent of batch size and serves to stabilize the feature distribution during training, thereby facilitating optimization and accelerating convergence. The ReLU activation function helps the model capture complex patterns while maintaining a smooth gradient flow.

#### 2.2.3. Multiscale Analysis ${(F}_{MS})$

EEG signals are complex and non-stationary; their attributes change over time and exhibit fractal-like properties, which necessitate their multiscale analysis to reveal multiple levels of brain activation, from rapid neuronal spiking to slower oscillations, and extract features. Traditional multiscale analysis techniques, such as the discrete wavelet transform (DWT) and the Fourier transform, do not adaptively capture the dynamic and multi-frequency features of EEG signals due to their predefined basis functions. As the brain activations are short and localized in time, we employ Multiscale Analysis mapping ${(F}_{MS})$ composed of temporal convolutions with several filters of various sizes to capture multiscale brain dynamics.

The mapping ${F_{MS}:R}^{1\times n\times F_{1}}\to R^{1\times (n/4)\times (3D_{1}\times F_{1})}$ receives $O^{2}{\in R}^{1\times n\times F_{1}}$ and processes it with three different operations, as shown in Figure 1, temporal multiscale convolution, concatenation, and average pooling:

(5) $$F_{MS}\left(O^{2}\right)=A\circ C\circ \phi_{MS}(O^{2}),$$

where $\phi_{MS}$, C and A represent multiscale temporal convolution, concatenation, and average pooling, respectively.

The multiscale temporal convolution $\phi_{MS}$ is defined as follows:

(6) $$\phi_{MS}\left(O^{2})=[\phi_{s1}(O^{2}),\phi_{s2}(O^{2}),\phi_{s2}(O^{2})\right],$$

where $\phi_{si}$, *i* = 1, 2, 3 are temporal convolution mappings with scales *si*, *i* = 1, 2, 3, which take $O^{2}$ in parallel and extract features across multiple scales using filters of different sizes, providing rich and discriminative information.

Each $\phi_{si}$ is specified as follows:

(7) $$O_{i}^{3}=\phi_{si}\left(O^{2};\;\theta_{si}^{\left(i\right)}\right)=B\circ R\circ \psi_{t}^{\left(i\right)}\left(O^{2}\right),\;i=1,\;2,\;3$$

where $\psi_{t}^{\left(i\right)}$ represents the ith temporal convolution, B is batch normalization, R is a ReLU activation function. Each $\psi_{t}^{\left(i\right)}$ is implemented as a depth-wise temporal convolutional layer (DTConv) with F1 filters of sizes *S_i_*, *i* = 1, 2, 3, and depth D. It drastically reduces the number of learnable parameters and computational cost, leading to better generalization with a small amount of training data. We used D = 2. The multiscale features $O_{1}^{3},O_{2}^{3},$ and $O_{3}^{3}$ produced by $\phi_{MS}$ are fused using concatenation to yield $O^{3}\in R^{1\times n\times \left(3D\times F_{1}\right)}$. Finally, the temporal dimension of $O^{3}$ is reduced by an average pooling layer with a window of size 1 × 4 and a stride of 4, yielding $O^{4}$ where $O^{4}{\in R}^{1\times (n/4)\times (3D_{1}\times F_{1})}$. The average pooling layer reduces model complexity, thereby avoiding overfitting. The $F_{MS}$ mapping computes the multiscale information in the form of multiscale spatiotemporal features and produces $O^{4}$ as an output, which is forwarded to the next block.

#### 2.2.4. High-Level Feature Learning ${(F}_{HLF})$

We design high-level feature learning mapping ${(F}_{HLF})$ to learn hierarchical and increasingly intricate and abstract features relevant to MDD. It is composed of one sequential convolution, one shortcut connection, and global average pooling. Mathematically, it is specified as follows:

(8) $$F_{HLF}\left(O^{4}\right)=A_{g}\left(\phi_{HLF}\left(O^{4}\right)⊕\psi_{p}\left(O^{4}\right)\right),$$

where $\phi_{HLF}$, ⊕ and $A_{g}$ denote sequential convolution, shortcut connection, and global average pooling; $\psi_{p}$ is a pointwise mapping to match the dimensions.

The sequential convolution $\phi_{HLF}$ is composed of two mappings $\phi_{sep}^{\left(i\right)},\;i=1,\;2$, specified as follows:

(9) $$\phi_{HLF}\left(O^{4}\right)=\;\phi_{sep}^{\left(2\right)}{\;\circ \;\phi }_{sep}^{\left(1\right)}\left(O^{4}\right)\;$$

where

(10) $$\phi_{sep}^{\left(i\right)}\left(O^{2i+2}\right)=B\circ R\circ \psi_{p}^{\left(i\right)}\circ B\circ R\circ \psi_{t}^{\left(i\right)}\left(O^{2i+2}\right),\;i=1,\;2$$

here $\psi_{t}^{\left(i\right)}$ and $\psi_{p}^{\left(i\right)}$ are depth-wise temporal convolution and pointwise convolution operations. The mapping $\phi_{sep}^{\left(i\right)}$ is implemented as a separable convolution block, where $\psi_{t}^{\left(i\right)}$, and $\psi_{p}^{\left(i\right)}$ in $\phi_{sep}^{\left(i\right)}$ blocks are implemented as depth-wise temporal convolution (DTConv) and pointwise convolution (PConv) layers. It learns intricate discriminative patterns associated with MDD while maintaining a low computational cost and parameter complexity, thereby avoiding overfitting. The shortcut connection is implemented as an element-wise addition ⊕, but the dimensions of $O^{4}$ and $O^{8}$ do not match, so first PConv is applied.

Finally, the mapping $A_{g}$ is realized through a global average pooling layer, which reduces the dimensionality of the feature space. It mitigates the curse of dimensionality and overfitting, thereby enhancing the model’s generalization capability.

#### 2.2.5. Classification Function ${(F}_{c})$

The features learned by the feature learning mappings described in the previous sub-sections are used to infer the label of the EEG segment to identify whether the subject is HC or suffers from MDD. For inference, the features are passed to the classification function $F_{c}$, which is implemented as a fully connected layer (FC) followed by a SoftMax layer. As there are two classes, HC and MDD, there are two neurons in the FC layer, which gives the activations *a* = [*a*_1_, *a*_2_]. The SoftMax layer converts the activations into the class probabilities *P* = [*p*_1_, *p*_2_], where

(11) $$p_{i}=\frac{e^{a_{i}}}{\sum_{j=1}^{2}e^{a_{j}}},\;i\;\in \left\{1,\;2\right\}.$$

### 2.3. Ensemble-like Classifier Based on MS-MDDNet

**Figure 2:**
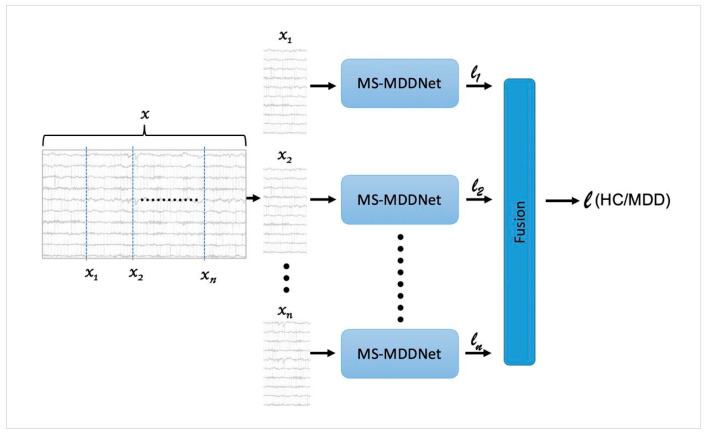
A prototype architecture of an ensemble learning classifier based on MS-MDDNet.

In machine learning, the core principle of an ensemble is to combine diverse predictions. Unlike conventional ensemble methods that rely on multiple distinct models, our approach leverages this principle by subdividing an inference-time EEG trial into sub-trials, treating each sub-trial as an independent observation, and fusing their outputs to form a robust final decision. The motivation stems from the fact that EEG signals are highly non-stationary and subject to noise [18]. By dividing an inference-time EEG trial (e.g., 10 s) into multiple sub-trials (e.g., 2 s) as shown in Figure 2, we capture diverse temporal patterns and reduce the risk of missing critical features. Treating each sub-trial as an independent observation, MS-MDDNet then predicts its label, and these predictions are fused to determine the final label:

(12) $${l=fuse(l}_{1},\;l_{2},\;\ldots ,\;l_{k}),$$

where l is the final predicted label of an inference-time EEG trial and $l_{1},l_{2},\;\ldots ,\;l_{k}\;$ represent the predicted labels of its sub-trials; and fuse is the fusion function, which is used to combine the predictions of sub-trials. The fusion function used to combine these predictions is majority voting, which is a widely adopted approach for ensemble decision-making.

The aggregation of sub-trial predictions mitigates local noise and improves stability. This strategy enhances generalization and reduces variance compared to relying on a single prediction per segment. Our reported results in Section 3.3 confirm that sub-trial-level voting improves accuracy and F1 scores.

Another motivation is to keep the MS-MDDNet architecture lightweight so that it can be trained with limited data without overfitting. The design of MS-MDDNet assumes that input EEG trials are short (e.g., 2 s), which helps keep the number of learnable parameters small and prevents overfitting when training data is limited. Since a short EEG trial (e.g., 2 s) may not accurately decode brain states (MDD or HC) due to the non-stationarity of EEG signals and noise, as demonstrated in Section 3.5, longer EEG trials (e.g., 10 s) are used during inference. If longer trials were used for both training and testing, the model complexity would increase, and the risk of overfitting would rise, since the number of available training trials would be small.

Furthermore, our approach eliminates the need to maintain multiple trained models, thereby avoiding storage issues.

Because our method does not strictly follow the conventional ensemble paradigm but instead relies on the ensemble principle—combining multiple predictions from different sub-trials to produce a final decision—we term it *an ensemble-like classification method*.

### 2.4. Training of MS-MDDNet

MS-MDDNet is modeled as a function f(.; θ): $\in R^{m\times n}$→ Y, which is composed of the mappings $F_{Pre},F_{S},$ $F_{MS},F_{HFL}\;and\;F_{C}$ as described in Equation (2), and the detail of each mapping is given in the previous subsections. The function involves the learnable parameters *θ* = { *θ_Pre_*, *θ_S_*, *θ_MS_*, *θ_HFL_*, *θ_C_*}, and learning of MS-MDDNet involves finding the best values $\hat{\theta }$ of these parameters using the training set, i.e., it is an optimization problem:

$$\hat{\theta }=\arg\underset{\theta }{\min}\sum_{i=1}^{N}L(y_{i},\;f\left(x_{i};\theta )\right)$$

where ${\left\{\left(x_{i},y_{i}\right)\right\}}_{i=1}^{N}$ is the training set and $L\left(y_{i},f\left(\;x_{i},\theta \right)\right)\;$ is the loss function. We used the cross-entropy loss.

To solve the problem, we employed ADAM optimizer, which learn the parameters *θ* using the following recursive equation:

(13) $$\theta_{t}=\theta_{t-1}-\alpha \frac{{\hat{r}}_{t}}{\sqrt{{\hat{s}}_{t}}+\varepsilon },$$

where

(14) $${\hat{r}}_{t}=\frac{r_{t}}{1-\beta_{1}^{t}},$$

(15) $${\hat{s}}_{t}=\frac{s_{t}}{1-\beta_{2}^{t}},$$

(16) $$r_{t}=\beta_{1}{\cdot r}_{t-1}+\left(1-\beta_{1}\right)\cdot g_{t},$$

(17) $$r_{t}=\beta_{1}{\cdot r}_{t-1}+\left(1-\beta_{1}\right)\cdot g_{t},$$

where *r* and *s* are the first and second moment estimates, respectively, and $g_{t}=\nabla_{\theta }L(\theta_{t})$ is the gradient of the loss function $L\left(\theta \right)=L\left(y_{i},f\left(\;x_{i},\theta \right)\right)$. The learning rate $\left(\alpha \right),{the\;exponential\;decay\;rates\beta }_{1}and\beta_{2}$ and epsilon (ε) are the hyperparameters. The moments *r* and *s* are initialized as zeros. In accordance with the optimization strategy proposed by Kingma and Ba [41], we configure the hyperparameters as follows: β_1_ = 0.9, β_2_ = 0.999, α = 0.001, and ε = 10^−8^. This configuration facilitates rapid network convergence, thereby enhancing the overall efficiency of the training procedure.

## 3. Experimental Results

### 3.1. Data Sets

The performance of MS-MDDNet and the ensemble-like classifier based on it was evaluated on three publicly available benchmark datasets (MODMA, MUMTAZ, and PRED + CT) to assess their robustness and generalizability across diverse data sources. This approach enables a comprehensive assessment of its effectiveness across contexts, helping uncover potential limitations that may not become evident when relying on a single dataset.

Although MODMA, MUMTAZ, and PRED + CT are relatively small compared to large-scale image or text datasets, they are among the most widely used and publicly available benchmark EEG datasets for MDD research. Using three distinct datasets ensures diversity in recording protocols, subject demographics, and hardware setups, thereby strengthening the generalizability of our findings.

To mitigate limitations in dataset size, we employed 10-fold CS-CV, a rigorous evaluation strategy that prevents subject-specific bias and overfitting. This approach provides a more reliable estimate of real-world performance compared to subject-dependent splits commonly used in prior studies.

EEG-based MDD detection is an emerging field, and large-scale standardized datasets are currently scarce. Our choice of datasets reflects the current state of the domain, and our method’s ability to achieve high accuracy across multiple datasets demonstrates robustness despite these constraints.

However, while dataset size is a limitation inherent to the field, our use of three independent datasets with rigorous CS-CV evaluation provides strong evidence of the proposed method’s effectiveness and generalization capability.

The subsequent paragraphs provide detailed descriptions of each dataset. All datasets were used exactly as provided in their respective repositories, with no additional preprocessing for artifact removal (e.g., EOG, EMG, blinks, ICA, or filtering).

#### 3.1.1. MODMA Dataset (DB1)

**Figure 3:**
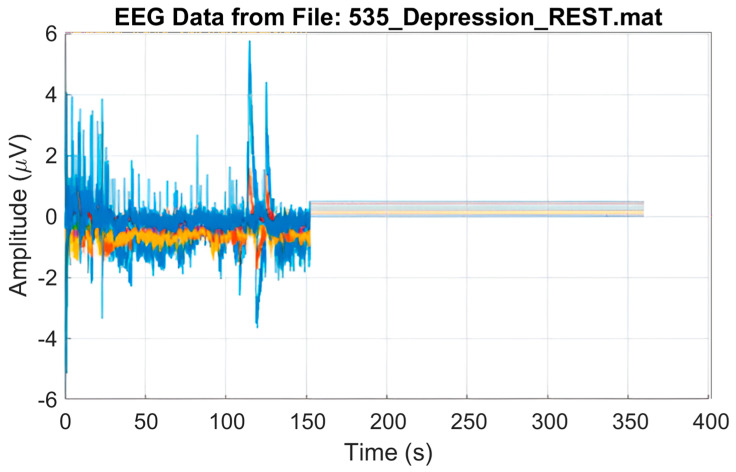
A corrupted recording of an excluded subject.

The MODMA dataset was acquired from 51 students at Lanzhou University [42]. Among the participants, 28 were HC, while 23 were diagnosed with MDD, with ages ranging from 16 to 56 years. EEG signals were recorded for a duration of five minutes under resting-state eyes-closed (EC) conditions using a 128-channel HydroCel Geodesic Sensor Net (HCGSN; Electrical Geodesics, Inc., Eugene, OR, USA) at a sampling rate of 250 Hz. Data acquisition was performed using Net Station software (version 4.5.4; Electrical Geodesics, Inc., Eugene, OR, USA). Electrode placement followed the international 10/10 system. In our experiments, three subjects (two with MDD and one healthy control, HC) were excluded due to corrupted recordings, as illustrated in Figure 3.

#### 3.1.2. MUMTAZ Dataset (DB2)

The dataset was collected by Mumtaz et al. [43] at the University of Sains Malaysia Hospital, Penang, Malaysia. It consists of recordings from 64 subjects aged between 27 and 54 years. Of these, 34 were diagnosed with MDD, while 30 were HC. EEG signals were acquired using a 19-channel electro-gel sensor EEG cap, with electrode placement following the international 10/20 system. A five-minute recording was obtained from each subject under EC resting-state conditions, sampled at 265 Hz. In our experiment, we used 29 MDD and 30 HC participants; 4 subjects were excluded due to poor recording, as shown in Figure 3.

#### 3.1.3. PRED + CT Dataset (DB3)

This dataset was obtained from the Patient Repository for EEG Data and Computational Tools, maintained by the University of New Mexico, Albuquerque, NM, USA [44]. It comprises EEG recordings from 121 participants, including 46 individuals diagnosed with major depressive disorder (MDD) and 75 healthy controls (HC). Recordings were conducted under eyes-closed (EC) resting-state conditions using 64 Ag/AgCl electrodes, with a sampling rate of 500 Hz. In our experiment, we excluded seven MDD files and two HC files: three files did not exist on the site, and six files had poor or corrupted signals.

### 3.2. Evaluation Procedure

To prevent data leakage and ensure that MS-MDDNet learns exclusively MDD-specific features while minimizing subject-related noise, the model was evaluated using 10-fold cross-subject cross-validation (CS-CV). The dataset was divided into ten stratified folds, each comprising data from distinct subjects. Ten experiments were conducted, with each iteration using one fold as the test set, another as the validation set, and the remaining eight folds as the training set. The model performance was reported as the average across 10 folds. The MODMA dataset contains data from 21 MDD subjects, which is fewer than the 27 HCs. To maintain a stratified fold distribution, 21 HCs were randomly selected to match the number of MDD patients.

Each subject’s EEG recording spans 5 min. For model training, we fixed the window size to 2 s and did not explore larger windows for two main reasons. First, the complexity of MS-MDDNet increases with window length; using longer windows would substantially increase model complexity. A 2-s window reflects the brain dynamics and provides the best balance between representational richness and model complexity. Second, the available datasets are relatively small, and increasing the window size would reduce the number of training segments, which in turn increases the risk of overfitting. However, for inference, we used a 10-s window size and an ensemble-like classifier to ensure the prediction is not influenced by momentary thoughts or distractions.

**Figure 4:**
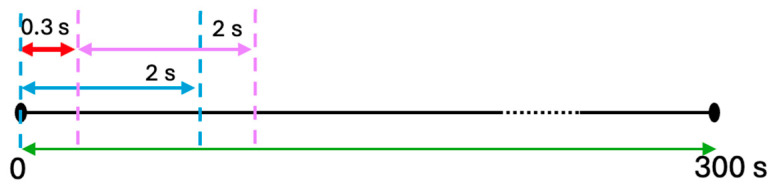
Training trials creation using an overlapping window; the red arrow represents the stride.

The 2-s window yields approximately 150 trials per subject and around 5700 training trials per fold. Despite MS-MDDNet’s lightweight architecture (34,812 LP), this sample size is insufficient for effective training without overfitting. To address this limitation, we increase the number of EEG trials with real data by employing a sliding window [45] with a 0.3-s stride for data augmentation, as illustrated in Figure 4. This technique increases the number of training trials from 5700 to approximately 36,000 within one fold.

During testing, a 10-s EEG trial is segmented into 2.0-s epochs. The ensemble classifier processes each epoch independently using the trained MS-MDDNet, and the final label for the trial is determined by fusing the individual predictions.

The MS-MDDNet model was implemented and trained using MATLAB’s Deep Learning Toolbox (R2022b). Training was performed on a single GPU using the Adam optimizer, as described in Section 2.4, with a piecewise learning rate strategy. The learning rate was initialized at 0.001, and a learning rate drop factor (LRDF) of 0.1 was used. The model was trained for a maximum of 25 epochs, with a batch size of 4 times the sampling rate and a validation patience of 5 as the stopping criterion. To mitigate overfitting and enhance generalization, L2 regularization was applied with a factor of 0.0001.

To evaluate the performance of the proposed model, widely recognized metrics were employed, i.e., accuracy (Acc), sensitivity (Sen), specificity (Spe), area under the receiver operating characteristic curve (AUC), and F1-score (F1). These measures are defined as follows:

(18) $$Acc=\frac{TP+TN}{TP+TN+FP+FN},\;$$

(19) $$Sen=\frac{TP}{TP+FN},\;$$

(20) $$Spe=\frac{TN}{TN+FP},$$

(21) $$F1=\frac{2\times TP\;}{2\times TP+FP+FN},\;$$

where *TP*, *TN*, *FP*, and *FN* stand for the number of true positives, true negatives, false positives, and false negatives, respectively.

### 3.3. The Performance of MS-MDDNet Across Three Public Datasets

Based on the ablation study on the MODMA dataset, the best configuration of MS-MDDNet was used to evaluate its performance on three public datasets. The results are shown in Table 2.

The results demonstrate the strong generalizability of MS-MDDNet across three benchmark EEG datasets. On the MODMA dataset, the model exhibits near-perfect performance (99.33% accuracy, 99.96% AUC) with small variability (±1.61% accuracy), indicating excellent stability and consistent classification. The PRED + CT dataset exhibits similarly high accuracy (96.61%) but slightly elevated variability (±5.46%), with a sensitivity-specificity trade-off (98% Sensitivity vs. 95.79% specificity). It suggests robust class separation (AUC 96.31%) despite potential data heterogeneity. The MUMTAZ dataset exhibits the highest performance variability (±11.99% accuracy) yet achieves perfect specificity (100%) and a high AUC (98.83%), indicating consistent identification of healthy controls and moderate fluctuations in sensitivity (95.69% ± 13.64%) in detecting MDD cases. The relatively lower performance on PRED + CT and MUMTAZ datasets may be attributed to MS-MDDNet’s configuration, which was tuned using the MODMA dataset.

Notably, all datasets achieve F1-scores greater than 95% and AUCs greater than 96%, confirming the model’s reliability across diverse populations and recording conditions. The minor performance variations likely stem from dataset-specific factors (e.g., participant demographics, EEG protocols), but the consistently high metrics underscore MS-MDDNet’s capacity to generalize. The near-perfect results on the MODMA dataset validate the architectural optimizations, while outcomes on the MUMTAZ and PRED + CT datasets validate the method’s resilience to real-world variability, establishing it as a clinically transferable solution for MDD screening.

### 3.4. Ablation Study

A series of experiments was conducted to assess the effectiveness of individual components within the model architecture and to determine the best values for their corresponding hyperparameters.

#### 3.4.1. The Effect of the Combination of Channels

**Figure 5:**
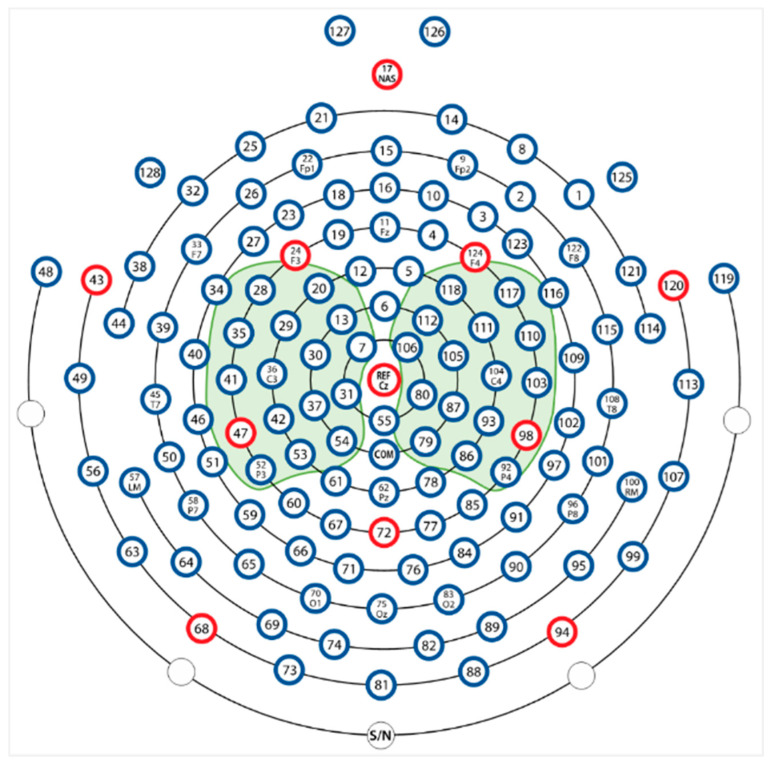
MODMA dataset; highlighted channels are selected from 128 channels.

EEG channels capture neural activity from distinct scalp locations. To investigate which brain regions, i.e., channels, are most informative for MDD detection, we conducted experiments using various channel combinations. These experiments employed the model configuration illustrated in Figure 1 and detailed in Table 1, with all activation functions replaced by the Swish function. The performance outcomes of eleven tested channel configurations are summarized in Table 3. The best channel configuration is the “Left and right central” (Option 9), achieving near-perfect accuracy (98.67% ± 3.12%) and F1-score (98.56% ± 3.47%), with the lowest variability. It indicates that the channels in the central brain region, as highlighted in Figure 5 [34], form the most discriminative and stable EEG signals for MDD detection.

#### 3.4.2. The Effect of Spatial Information Integration

The $F_{pre}$ block aggregates informative EEG channels from distinct brain regions across both hemispheres. Within each region, the selected channels were grouped and subsequently passed to a convolutional layer, enabling the model to learn localized patterns that contribute to MDD classification. All experiments employed the configuration described in Table 1 and depicted in Figure 1, except that all activation functions were replaced with the Swish function. Three experimental configurations were tested. The results, presented in Table 4, demonstrate the significance of the $F_{pre}$ module within the MS-MDDNet architecture, notably improving classification accuracy when convolutional processing is applied, compared to configurations that use average pooling or independent channel processing.

#### 3.4.3. The Effect of Normalization Techniques

To investigate the impact of different normalization techniques on model performance, three approaches—Batch Normalization (BN), Layer Normalization (LN), and Grouped Normalization (GN)—were evaluated within distinct modules of the MS-MDDNet architecture. All other architectural components and hyperparameters were held constant, as specified in Figure 1 and Table 1, with Swish activation functions applied throughout. The results, summarized in Table 5, demonstrate that employing GN within the spatial analysis module and BN in all other modules yields the highest classification performance. In contrast, BN yielded the lowest performance across all blocks, reducing it by 8.14%.

#### 3.4.4. The Effect of Different Activation Functions

To investigate the influence of activation functions on MS-MDDNet performance, five widely recognized functions were tested within the MS-MDDNet architecture. The experimental setup followed the configuration outlined in Table 1 and Figure 1, with activation functions being the only variable. As reported in Table 6, ReLU improved the performance by 0.67% compared to the others, due to its computational efficiency, sparse activation characteristics, and its ability to mitigate the vanishing gradient problem by propagating significant positive features while zeroing out non-positive inputs. The Tanh function exhibited the lowest performance.

#### 3.4.5. The Effect of the Number of Filters in the Spatial Analysis Module

To determine the best number of filters in the spatial analysis module, a series of experiments was conducted using the MS-MDDNet configuration depicted in Figure 1 and detailed in Table 1. The number of spatial convolution filters (F1) was varied, while all other parameters were held constant. The results, summarized in Table 7, indicate that setting F1 = 8 yields the highest classification accuracy. Reducing F1 to 4 resulted in a slight decline in performance due to underfitting, caused by insufficient capacity to model complex patterns. Conversely, increasing F1 to 12 led to overfitting, as the expanded model capacity captured redundant or noisy patterns, thereby diminishing accuracy.

#### 3.4.6. The Effect of the Multiscale Analysis Module

To evaluate the impact of multiscale temporal analysis on MS-MDDNet’s performance, experiments were conducted using the configuration outlined in Figure 1 and Table 1, with modifications applied only to the multiscale analysis module. As summarized in Table 8, the absence of this module resulted in diminished performance, indicating its critical role in capturing relevant temporal features. Notably, the use of three distinct temporal scales yielded the highest classification accuracy, emphasizing the effectiveness of multiscale feature representation in improving model generalization.

Furthermore, to determine the optimal number of convolutional filters within each temporal scale of the multiscale analysis module, a series of experiments was conducted. The results, presented in Table 9, indicate that employing 125, 62, and 31 filters across the three respective scales yields the highest classification performance. In contrast, reducing the number of filters led to a substantial drop in accuracy, exceeding 10%, suggesting insufficient representational capacity to capture discriminative temporal features.

#### 3.4.7. The Effect of the High-Level Feature Learning Module

To evaluate the contribution of the hierarchical feature learning module ($F_{HFL}$) and its constituent layers, a series of experiments was conducted. The results, presented in Table 10, demonstrate a marked decline in model performance when $F_{HFL}$ is excluded, underscoring its critical role in effective MDD detection. Furthermore, the inclusion of two temporal separable convolution blocks and a skip convolution significantly enhances performance. The temporal separable convolutions facilitate the extraction of rich, hierarchical temporal features, while the skip connection mitigates overfitting and the vanishing gradient problem, thereby ensuring the extraction of MDD-relevant features.

#### 3.4.8. The Effect of Pooling Layers

MS-MDDNet incorporates two pooling layers, one within $F_{MS}$ and another in $F_{HFL}$, to reduce computational complexity and enhance representational efficiency. To examine the impact of different pooling strategies, experiments were conducted with three configurations: max pooling (MP) and global max pooling (GMP), average pooling (AP) and GMP, and AP and global average pooling (GAP). All other architectural and hyperparameter settings adhered to the specifications outlined in Figure 1 and Table 1, with the pooling method being the only variable. As presented in Table 11, the results demonstrate that average pooling consistently outperforms max pooling, attributing to its superior capacity for distributed feature representation, enhanced generalization, and reduced sensitivity to noise. Unlike max pooling, which extracts only the peak values and may amplify discontinuities and noise, average pooling integrates continuous activations across channels, ensuring a more comprehensive feature aggregation.

The ablation experiments systematically revealed the importance of key components of the MS-MDDNet. Using left and right central electrodes, it achieved the best accuracy (98.67% ± 3.12%) and F1-score (98.56% ± 3.47%), outperforming all other configurations. The spatial analysis module significantly enhanced its performance. For normalization, GN in the spatial analysis module, combined with BN elsewhere, yielded the best results, while LN performed poorly. Among activation functions, ReLU proved superior due to computational efficiency and mitigation of vanishing gradients, outperforming Swish and Tanh.

Further, tuning revealed that eight filters in the spatial analysis module provided the best model capacity, with fewer filters resulting in underfitting and more leading to overfitting. The multiscale temporal module was critical for performance; using three temporal scales with 125, 62, and 31 filters, respectively, maximized accuracy. The hierarchical feature learning module with skip connections is indispensable, as it enhances temporal feature extraction and prevents overfitting. Finally, average pooling consistently surpassed max pooling across modules due to its robustness to noise and ability to capture distributed activations. Collectively, these components and hyperparameters yielded a highly accurate and efficient architecture for MS-MDDNet in MDD detection.

### 3.5. The Robustness of MS-MDDNet and Ensemble-like Classifier

In this section, we address three key questions: (1) is the method robust; (2) is an ensemble-like classifier effective; and (3) is there overfitting?

To address the first question, the 10-fold cross-validation results for the MODMA dataset are presented in Table 12, which demonstrate exceptional consistency and robustness in the detection capability of the method. Eight out of ten folds achieved the best performance (Folds 1, 3–9), while the lowest-performing fold (Fold 2) still attained high results (95% accuracy, 90% sensitivity, 100% specificity). All folds achieved near-perfect AUC values, confirming that the method performs well in discriminating between classes. Fold 10 showed a minor reduction in specificity (96.67%), but overall metrics remained outstanding (98.33% accuracy, 98.36% F1-score). The negligible standard deviation across folds highlights the method’s stability and robustness. It is important to note that, in each fold, the training, validation, and test sets contain data from different subjects; this procedure substantially reduces the likelihood of data leakage.

To address the second question, the 10-fold cross-validation results of both the ensemble model and the single MS-MDDNet are presented in Table 13. The results indicate that the ensemble classifier outperforms the single model and demonstrates greater robustness, as reflected by the lower standard deviation across all metrics. This is because, in the ensemble approach, errors made by MS-MDDNet on certain segments are compensated for by other segments, thereby reducing both bias and variance.

Regarding the third question on overfitting, despite the high performance observed in Table 12, we consider the risk of overfitting to be minimal for two reasons. First, the method was evaluated using CS-CV; this ensures the model is tested on unseen subjects, thereby preventing it from learning subject-specific noise. Second, the high accuracy is attributed to the ensemble-like structure of the classifier (as explained above), which improves generalization rather than simply memorizing the training data. However, we acknowledge that the dataset is relatively homogeneous, which yields high within-dataset separability but may not fully reflect real-world heterogeneity. Therefore, the near-perfect results should be interpreted primarily as within-dataset performance, which is a limitation of the current study.

**Figure 6:**
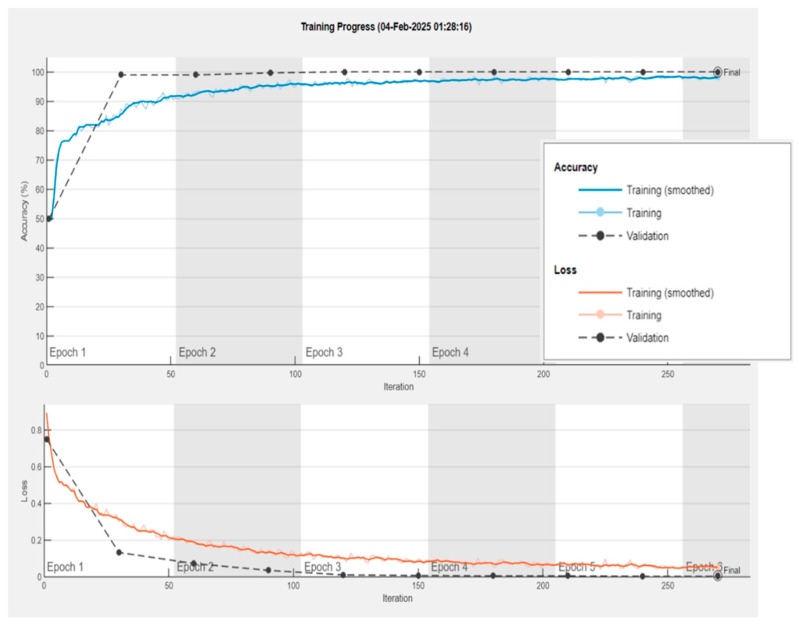
Training and validation curves of MS-MDDNet.

Second, overfitting is typically characterized by a widening gap between training and validation curves as training progresses. However, Figure 6 illustrates that the gap between the training and validation curves progressively narrows, and the algorithm terminates once the two curves converge closely. This convergence indicates that the model generalizes well and does not exhibit signs of overfitting.

**Figure 7:**
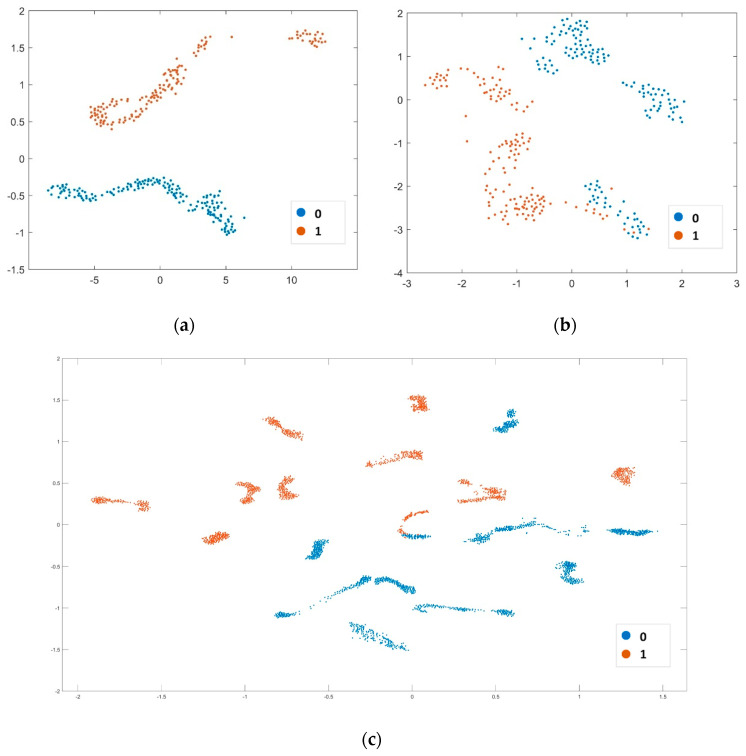
The t-SNE plot of (**a**) the best fold, (**b**) the worst fold, and (**c**) the features extracted from 10 folds, where 1 represents MDD and 0 represents HC.

Furthermore, we qualitatively illustrated that the learned features are highly discriminative. To demonstrate this, we extracted 48 learned features from the GAP layer of the test sets for the best fold, worst fold, and all folds and visualized them using t-Distributed Stochastic Neighbor Embedding (t-SNE), as shown in Figure 7a–c. The visualization of 48-dimensional features projected via t-SNE reveals near-complete separation between MDD (class 1) and HC (class 0) in the best fold (Figure 7a), illustrating the model’s capacity to extract neurophysiological distinct patterns. Even in the worst fold (Figure 7b), separation remains strong, with minimal overlap confined to a small cluster in the bottom right; this is consistent with the fold’s slight sensitivity drop. Notably, the concatenated features across all 10 folds (Figure 7c) exhibit robust clustering with no significant class mixing, highlighting the consistency of the learned representations across data partitions. The two key strengths are obvious. First, the clear separation across folds indicates that MS-MDDNet captures discriminative EEG signatures specific to MDD, likely reflecting altered brain dynamics (e.g., asymmetric frontal activity or aberrant oscillatory patterns). Second, minimal overlap in the worst fold and preserved separation in the concatenated features illustrate the model’s resilience to data variability.

### 3.6. Comparison with the SOTA Methods

A summary of the comparison between the MS-MDDNet-based method and SOTA methods across the three datasets is given in Table 14.

The MS-MDDNet-based method demonstrates a balance between computational efficiency, data representativeness, and classification performance compared to SOTA methods across MODMA, MUMTAZ, and PRED + CT datasets. Unlike complex architectures such as that by Zhang et al. (2023) [29], which utilizes a fusion of RNN, LSTM, and 2D-CNN with high time and space complexity, or the model developed by Gan et al. (2025) [36] using 3D-CNN, or that by Xu et al. (2024) [27], which is a hybrid of GCN and 2D-CNN with high spatial complexity, MS-MDDNet leverages a streamlined 1D-CNN design. It also contrasts with Xia et al. (2023) [28], which relies on multi-head self-attention but ignores temporal dynamics, thereby limiting generalizability. The architecture of MS-MDDNet significantly reduces the number of learnable parameters and avoids excessive computational overhead, making it both time- and memory-efficient.

MS-MDDNet demonstrates robust channel efficiency across multiple EEG datasets, outperforming or matching SOTA methods with significantly fewer input channels. On MUMTAZ, it achieves 96.21% accuracy using only 2 channels, compared to the 20 channels required by Zhou et al. (2024) [33] or 19 channels used by Gan et al. (2025) [36] and Wang et al. (2025) [30] for comparable results. Similarly, on MODMA, MS-MDDNet uses 32 channels, compared with 64 in Zhang et al. (2023) [29] and 128 in Xu et al. (2024) [27], while on PRED + CT it uses only 6 channels (Xu et al. did not specify the channel count). This lightweight configuration substantially reduces hardware complexity and setup requirements, addressing limitations in existing high-density systems, such as those with 128-channel montages or limited channel attribution (e.g., Xu et al., 2024 [27]), thereby enhancing feasibility in wearable, resource-constrained, and clinical neurodiagnostic environments.

MS-MDDNet consistently delivers high performance across key classification metrics, including accuracy, specificity, F1 score, and AUC, while maintaining low architectural complexity and interpretability. On the MODMA dataset, it achieved 99.33% accuracy using 32 channels, outperforming Zhang et al. (2023) [29] with 93.77% (64 channels), Carlle et al. (2023) [39] with 50.9%, and Xu et al. (2024) [27] with 90.56%. Similarly, on PRED + CT, MS-MDDNet slightly outperformed Xu et al. (2024) [27], achieving 96.61% accuracy versus 96.51%, along with stronger specificity (98.00%) and F1 score (95.75%). MS-MDDNet outperforms Wang et al. (2025) [30] by improving accuracy by ~15%. On MUMTAZ, although its 96.21% accuracy is modestly lower than Zhou et al. (2024)’s [33] 98.59%, MS-MDDNet uses only 2 channels instead of 20 and achieves perfect AUC (100%), indicating optimal class separability. Unlike Carlle et al. (2023) [39], who rely on synthetic data, and other SOTA methods such as Xia et al. (2023) [28] and Zhou et al. (2024) [33], which emphasize attention mechanisms or custom activations at the expense of transparency, MS-MDDNet preserves interpretability while excelling across metrics.

Moreover, MS-MDDNet avoids the pitfalls associated with the use of synthetic data and excessive preprocessing, as noted in studies such as Carlle et al. (2023) [39], which exhibited poor generalizability and chance-level performance with artificial datasets. The proposed method enhances robustness through real-data augmentation via overlapping sliding windows, a strategy that maintains EEG signal integrity while expanding the training set.

The complexity of a deep learning model is measured by the number of learnable parameters. The more learnable parameters the model has, the more complex it becomes, and the greater the chances of overfitting when the available labeled data is limited. The last column of Table 1 lists the number of learnable parameters for each model module, and the total is 34,812. Deep learning-based models in SOTA methods involve hundreds of thousands of learnable parameters; for example, the model by Xia et al. [28] has 3,295,378 parameters. Given this, the proposed model is surely lightweight.

Overall, the lightweight MS-MDDNet-based ensemble classifier has shown superiority in three interlinked dimensions: model simplicity, channel economy, and generalization performance. It consists of a small number of learnable parameters due to its simple architecture, which includes separable convolutions. The model avoids overfitting by following a cross-subject evaluation procedure. Furthermore, the MS-MDDNet interprets the decision-making process and feature learning capabilities, as well as the influence of brain rhythms. Its adaptability across multiple datasets, minimal reliance on preprocessing, and its interpretability position it as an effective and scalable solution for EEG-based MDD detection.

## 4. Discussion

There are several advantages of MS-MDDNet. First, it has a lightweight architecture that can be trained using a limited amount of data, thereby minimizing the risk of overfitting. Second, it learns discriminative features that are relevant to MDD and healthy controls. Third, its predictions are interpretable and explainable. Fourth, it strikes a good balance between complexity and performance, producing results comparable to those of state-of-the-art methods with significantly less complexity.

### 4.1. The Effect of Brain Waves on MDD Detection

EEG signals are usually decomposed into five frequency bands (brain waves or rhythms)—Delta (0.5–4 Hz), Theta (4–8 Hz), Alpha (8–13 Hz), Beta (13–30 Hz), and Gamma (30–100 Hz), each associated with distinct neural activities. Prior studies differ in the extent to which band-related features are associated with MDD: alpha [46,47,48] and beta [4,49,50]. To examine the frequency-specific contribution to MS-MDDNet’s decision-making process, test set EEG trials were separately filtered into individual waveforms and fed into the pre-trained MS-MDDNet model to infer their corresponding labels. The classification results for each frequency band are reported in Table 15, Table 16 and Table 17, which reflect the model’s performance across three experimental conditions: best-performing fold, worst-performing fold, and the average across ten cross-validation folds.

The gamma band unequivocally emerged as the most salient neurophysiological biomarker for MDD detection. It exhibited near-perfect performance across all evaluation metrics, including 100% accuracy, sensitivity, specificity, AUC, and F1-score, in both the best- and worst-performing folds. When averaged across 10 cross-validation folds, gamma-band features continued to yield exceptional classification performance (95% accuracy, 100% sensitivity, and 96.67% F1-score). These findings are corroborated by prior studies [34,51,52,53], which substantiate gamma’s relevance in MDD detection and reinforce the biological plausibility of MS-MDDNet. This prominence aligns with gamma activity’s established role in modulating mood, cognition, and emotional regulation, which are notably disrupted in MDD.

In contrast, other bands showed limited utility. Alpha, Beta, and Theta failed to detect MDD cases (0% sensitivity across best- and worst-performing folds), with accuracy near chance (~50%). Delta displayed moderate but unstable performance (73–100% accuracy), with low specificity (50–64%) and inconsistent performance across folds.

These results emphasize that the gamma band is the primary EEG biomarker for MDD detection, while slower bands lack reliable diagnostic relevance. This insight prioritizes gamma-focused analysis for future depression diagnostics and aligns with established neurophysiological studies of MDD.

### 4.2. The Correlation of Features with the Gamma Band

**Figure 8:**
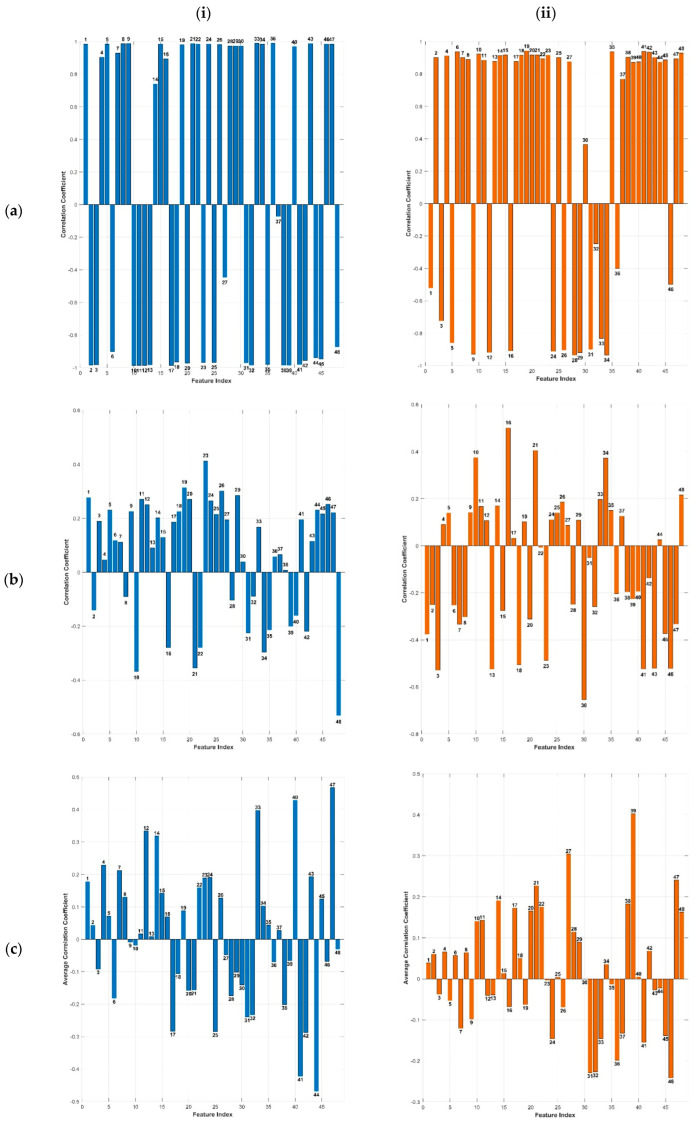
The correlation of learned features with Gamma Energy, (**a**–**c**) for the best, the worst, and the average across 10-folds, left (**i**) and right (**ii**) columns correspond to HC and MDD, respectively.

The findings from the preceding section indicate that the gamma band serves as the primary EEG biomarker, influencing MS-MDDNet’s decision-making process for detecting MDD. In this section, we investigate the relationship between the learned features and gamma energy to determine how the gamma-band correlates with the high-level features learned by the model. Figure 8 illustrates the correlation profiles for the best, worst, and average results across 10 folds of a 10-fold cross-validation, highlighting the relationship between the gamma band and the learned features for both the MDD and HC classes.

The magnitude and sign of the correlation coefficient *r* indicate the nature of the relationship between variables. A positive or negative value denotes the direction of the correlation, whereas its absolute value reflects the strength: strong correlation (*r* ≥ 0.7), moderate correlation (0.7 > *r* ≥ 0.3), and weak correlation (0.3 > *r* ≥ 0).

For MDD subjects, gamma energy shows predominantly positive correlations across features. In the best-performing fold, 65% (31/48) of features exhibited strong positive correlations (*r* ≥ 0.7), with one feature showing moderate correlation. This pattern persists across the average of 10 folds, where more than 50% of features remain positively correlated (including 4 strong correlations), although the worst fold shows weaker positive associations. Conversely, HC subjects demonstrate divergent behavior: the best fold splits features evenly between strong positive (50%) and negative correlations (50%), while both the worst fold and 10-fold average show predominantly moderate positive correlations between 33 and 25 features, respectively. Critically, more than 50% of features display opposite correlation directions between MDD and HC in the average of 10 folds (e.g., positive in MDD but negative in HC, or vice versa). This opposition highlights their discriminative power for classification, with nine features consistently positive for MDD and twelve for HC across folds. The MS-MDDNet leverages the correlation between gamma energy and the learn [34,51,52,53] features to distinguish between groups. However, subject-specific variability in the CS-CV protocol may influence the correlation strengths. Overall, gamma energy correlates more strongly and uniformly with features in MDD, whereas HC shows greater heterogeneity, with notable negative correlations in high-performing folds.

### 4.3. Interpretability of Predictions

Following the analysis of correlation plots derived from the best-performing fold, the worst-performing fold, and the average across ten folds (as shown in Figure 8), it was observed that Features 10 and 48 exhibit a consistent pattern: both are negatively correlated with gamma energy in HC and positively correlated in individuals with MDD across all three scenarios. This consistent divergence suggests that these features can serve as effective biomarkers for interpreting the decision-making process of MS-MDDNet.

**Figure 9:**
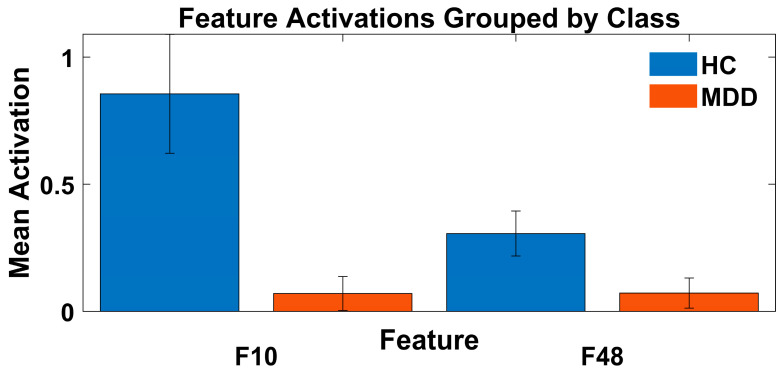
The Average activations of features 10 and 48 across 10 folds.

To further validate their discriminative capacity, we computed the mean and standard deviation of these two features, as illustrated in Figure 9. The figure reveals that the range of values for Features 10 and 48 in HC and MDD do not overlap. This separation further reinforces the notion that these features can reliably distinguish between the two classes. Given their respective value ranges and correlations with gamma energy, the MS-MDDNet’s predictions of a subject’s state from their EEG trial (MDD or HC) can be considered reliable. This interpretability is based on their consistent negative or positive correlation with gamma energy, which plays a crucial role in MDD detection, as demonstrated in our findings in Section 4.1 and corroborated by previous studies [34,51,52,53].

While the gamma band has been associated with MDD, it is not an exclusive or definitive marker. EEG signals are complex and influenced by multiple frequency bands, spatial patterns, and temporal dynamics. Relying solely on gamma band power can lead to oversimplification and reduced diagnostic accuracy. Our model does not merely detect the gamma band; it learns multiscale spatial-temporal representations across all relevant frequency bands. The observed correlation with gamma energy validates the model’s interpretability, but the classification decision is based on a richer feature set that captures subtle patterns beyond gamma. Gamma band characteristics can vary significantly across individuals and recording conditions. Our approach mitigates this variability by leveraging deep learning to extract robust features and by aggregating across sub-trials, improving reliability compared to a single-band heuristic.

The correlation analysis is provided to enhance interpretability, not to imply that gamma alone drives the decision. By showing that learned features align with known biomarkers, we increase clinician confidence in the model while still benefiting from the complex, non-linear feature interactions that deep learning captures.

In short, gamma-band correlation supports interpretability, but the proposed approach is essential because it combines multiple discriminative features and thereby improves robustness.

### 4.4. Clinical Relevance and Implications

Major Depressive Disorder (MDD) affects over 322 million people worldwide and remains difficult to diagnose due to reliance on subjective assessments. Electroencephalography (EEG) provides a promising, non-invasive tool for diagnosing MDD. However, robust and explainable diagnosis requires analyzing the broader spatiotemporal dynamics of EEG signals.

The proposed MS-MDDNet framework addresses these limitations by extracting multiscale spatial-temporal features from EEG signals, enabling more comprehensive and accurate detection. Importantly, the model achieves high accuracy using a minimal number of EEG channels, reducing hardware costs and setup complexity—critical for outpatient clinics and telemedicine applications. Furthermore, the model’s interpretability is enhanced through correlation analysis with gamma-band energy, bridging the gap between deep learning and clinically recognized biomarkers. This transparency fosters clinician trust and supports integration into diagnostic workflows.

Robustness is another key clinical advantage. EEG recordings are often affected by noise and subject variability. By employing an ensemble strategy across subsegments, MS-MDDNet mitigates these issues and improves reliability in real-world settings. The model’s strong performance across three independent datasets further demonstrates its generalization capability, which is essential for deployment in diverse clinical environments.

Beyond diagnosis, the approach has broader implications for continuous monitoring and personalized treatment planning. Integration with wearable EEG devices could enable home-based assessments, reducing healthcare burdens and improving accessibility for patients in remote areas. Objective biomarkers derived from EEG can complement psychometric evaluations, enabling more precise, data-driven clinical decisions.

Overall, MS-MDDNet is not merely a technical advancement but a clinically relevant solution that combines accuracy, interpretability, and scalability.

### 4.5. Limitations and Future Work

Despite MS-MDDNet’s excellent performance, we acknowledge several limitations. First, the current model does not classify MDD severity levels (e.g., mild, moderate, severe), which is clinically important for guiding treatment strategies. Second, generalizability may be limited due to the relatively small size of the publicly available datasets (MODMA, MUMTAZ, PRED + CT) used in the field compared to those in large-scale clinical repositories. Third, the correlation analysis was restricted to the gamma band, despite evaluating all frequency bands, due to its established relevance to MDD, and provided valuable insights. Excluding correlation patterns in other frequency bands limits interpretability and constrains the analysis to the frequency domain. Expanding to spatial and temporal attribution methods could yield a deeper understanding of the neural mechanisms underlying MDD.

Also, we trained and evaluated MS-MDDNet using cross-subject cross-validation (CS-CV), ensuring that no information from the test subjects was present in the training set. This setup allows the model to be assessed on individuals entirely unseen to it, providing a realistic approximation of independent external validation. However, we acknowledge that CS-CV cannot fully replace true external validation on data collected from different sites or acquisition conditions.

Future work will extend MS-MDDNet to classify MDD severity levels, validate performance in large-scale multi-center trials, and explore real-time deployment in telehealth platforms. To further enhance accuracy and interpretability, we plan to integrate mechanisms such as self-attention. Beyond frequency-domain correlation, intrinsic interpretability strategies—such as spatial attribution maps, temporal attention, multi-band feature disentanglement, interpretable convolutional filters, and prototype-based learning—can provide richer insights. Incorporating these methods will expand interpretability from spectral analysis to spatial-temporal dynamics, strengthening both clinical trust and scientific understanding.

We did not address subjects with comorbid conditions such as anxiety or bipolar disorder because the publicly available datasets used in this study do not include them. Addressing such conditions would require access to datasets that explicitly contain these scenarios, either through future public releases or through our own data collection efforts. This is also an important direction of future work.

In its current state, however, MS-MDDNet already represents a powerful and efficient tool that can support neurologists in the timely and accurate diagnosis of MDD.

## 5. Conclusions

In this study, we introduced MS-MDDNet, a lightweight, end-to-end deep learning model and an ensemble-like method based on it for effective MDD detection from EEG signals. The model architecture, which leverages a combination of spatial and temporal convolutions, average pooling, and separable convolution, demonstrates a powerful capacity for extracting dominant neurophysiological features while maintaining low computational complexity. The analysis of the performance of the model across well-known frequency bands confirms that the gamma band is the most salient and discriminative biomarker for MDD detection. Furthermore, correlation analysis between gamma energy and the features learned by MS-MDDNet revealed that specific learned features, notably Features 10 and 48, exhibited consistent, opposing correlations with gamma energy across the MDD and HC groups, indicating that these features explain the model’s predictions and make them transparent.

The robustness and generalization capabilities of MS-MDDNet were rigorously validated across three distinct, publicly available benchmark datasets using the CS-CV protocol. This protocol ensures that there is no overfitting through splitting the data by subjects, such that the data of a subject appears in either the training or test set. The model achieved accuracies of 96.21% on the MUMTAZ dataset and 96.61% on the PRED + CT data, which are comparable to those of the state-of-the-art (SOTA) methods. It achieved 99.33% accuracy on the MODMA dataset, improving upon all SOTA methods by 9%. Further, the model achieves these accuracies despite having much less model complexity and using a small number of EEG channels compared to SOTA methods. These results establish the superiority of the MS-MDDNet-based method over current SOTA methods, not only in performance but also in architectural efficiency, as it requires significantly fewer EEG channels and fewer learnable parameters. These characteristics of the model help it avoid overfitting and the high computational resource demands, which are the potential pitfalls of more complex models or those reliant on synthetic data. The model’s robustness and the interpretability of its predictions make it a valuable tool for supporting clinicians and individuals in diagnosing MDD with confidence, thereby enhancing transparency in decision-making and strengthening clinical credibility.

## Figures and Tables

**Table 1:** The Specification of MS-MDDNet: KS (Kernel Size), KN (Number of Kernels), GN (Group Normalization), BN (Batch Normalization), ACT (Activation Function), #LPs (Number of Learnable Parameters), SConv (Spatial Convolution), DTConv (Depth- wise Temporal Convolution), AP (Average Pooling), PConv (Pointwise Convolution), GAP (Global Average Pooling), FC (Fully Connected Layer), and SR (Sampling Rate).

Block	Layer	KS	KN	Input Size	Output Size	BN/GN/ACT	#LPs
$F_{pre}$	Input	–	–	32 × 500 × 1	32 × 500 × 1	–	–
	SConvᵣ	1 × 16	1	16 × 500 × 1	1 × 500 × 1	–	17
	SConvᵢ	1 × 16	1	16 × 500 × 1	1 × 500 × 1	–	17
	Concatenation	–	–	2 × (1 × 500 × 1)	2 × 500 × 1	–	–
$F_{S}$	SConv	8 × 1	F_1_ = 8	2 × 500 × 1	1 × 500 × 8	GN/ReLU	40
$F_{MS}$	DTConv (S_1_ = SR/2 = 125)	1 × S_1_	D_1_ = 2	1 × 500 × 8	1 × 500 × 16	BN/ReLU	2048
	DTConv (S_2_ = SR/4 = 62)	1 × S_2_	D_1_ = 2	1 × 500 × 8	1 × 500 × 16	BN/ReLU	1040
	DTConv (S_3_ = SR/8 = 31)	1 × S_3_	D_1_ = 2	1 × 500 × 8	1 × 500 × 16	BN/ReLU	544
	Concatenation	–	–	3 × (1 × 500 × 16)	1 × 500 × 48	–	–
	AP	1 × 4	–	1 × 500 × 48	1 × 125 × 48	–	–
$F_{HFL}$	DTConv (S_3_)	1 × S_3_	D_1_ = 2	1 × 125 × 48	1 × 125 × 96	BN/ReLU	3264
	PConv	1 × 1	F_3_ = 96	1 × 125 × 96	1 × 125 × 96	BN/ReLU	9504
	DTConv (S_3_)	1 × S_3_	D_1_ = 2	1 × 125 × 96	1 × 125 × 192	BN/ReLU	6528
	PConv	1 × 1	F_2_ = 48	1 × 125 × 192	1 × 125 × 48	BN/ReLU	9360
	PConv	1 × 1	F_2_ = 48	1 × 125 × 48	1 × 125 × 48	–	2352
	GAP	–	–	1 × 125 × 48	1 × 1 × 48	–	–
$F_{C}$	FC	–	–	1 × 1 × 48	1 × 1 × 2	SoftMax	98
Total Learnable Parameters						34,812	

**Table 2:** The performance of MS-MDDNet across three public datasets.

Dataset	ACC%	Sen%	Spe%	AUC%	F1%
MUMTAZ	96.21 ± 11.99	95.69 ± 13.64	100 ± 00	98.83 ± 03.48	97.25 ± 08.70
PRED + CT	96.61 ± 05.46	98.00 ± 05.26	95.79 ± 05.99	96.31 ± 04.84	95.75 ± 06.75
MODMA	99.33 ± 01.61	99.00 ± 03.16	99.67 ± 01.05	99.96 ± 00.14	99.31 ± 01.69

**Table 3:** The performance of MS-MDDNet using different combinations of channels.

Options	Channels Names	ACC%	F1%
1	Fp1, Fp2	70.07 ± 31.23	63.20 ± 40.78
2	Fp1, Fp2, T7, T8	81.91 ± 30.48	75.79 ± 40.45
3	½ (Fp1 + T7), ½ (Fp2 + T8)	87.38 ± 17.34	81.33 ± 28.12
4	Fp1/Fp2, F3/F4, C3/C4, O1/O2, F7/F8, T7/T8, P7/P8	91.00 ± 15.66	86.15 ± 25.82
5	1/3(Fp1 + F3 + F7),1/3 (Fp2 + F4 + F8),C3,C4,O1,O2,½ (T7 + P7),½(T8 + P8)	94.68 ± 15.73	96.32 ± 10.48
6	1/7(Fp1 + F3 + F7 + C3 + O1 + T7 + P7), 1/7(Fp2 + F4 + F8 + C4 + O2 + T8 + P8),	89.36 ± 17.51	83.98 ± 31.57
7	All left and right	95.50 ± 08.01	96.15 ± 06.68
8	Left and right temporal	93.67 ± 15.57	95.41 ± 10.39
9	**Left and right central**	**98.67 ± 03.12**	**98.56 ± 03.47**
10	Left and right frontal	96.50 ± 06.87	96.97 ± 05.82
11	Left and right parietal–occipital	97.17 ± 06.24	96.74 ± 07.48

**Table 4:** The impact of spatial information integration ${(F}_{pre})$ module.

Experiment	ACC%	Sen%	Spe%	AUC%	F1%
Without $F_{pre}$	93.71 ± 14.30	100 ± 00	87.82 ± 27.51	99.00 ± 02.92	95.23 ± 10.61
With $F_{pre}$ avg	86.24 ± 17.64	81.67 ± 33.78	90.33 ± 23.54	95.56 ± 11.17	80.87 ± 31.44
With $F_{pre}$ conv	**98.67 ± 03.12**	**97.67 ± 06.30**	**99.67 ± 01.05**	**100 ± 00**	**98.56 ± 03.47**

**Table 5:** The effect of normalization techniques on the performance of MS-MDDNet; T denotes type.

Block	T	ACC%	Sen%	Spe%	AUC%	F1%
All Blocks	BN	90.63 ± 14.58	87.00 ± 26.59	94.52 ± 16.21	99.44 ± 01.50	87.84 ± 22.42
All Blocks	GN	94.83 ± 12.38	92.33 ± 24.24	97.33 ± 08.43	98.21 ± 03.77	92.61 ± 19.60
$F_{S}$		**98.67 ± 03.12**	**97.67 ± 06.30**	**99.67 ± 01.05**	**100 ± 00**	**98.56 ± 03.47**
$F_{MS}$		95.48 ± 10.17	93.67 ± 20.03	97.00 ± 09.49	98.29 ± 05.41	94.06± 14.78
All Blocks	LN	97.85 ± 04.16	97.33 ± 08.43	98.39 ± 02.72	99.63 ± 00.93	97.65 ± 04.77
$F_{S}$		94.19± 13.15	94.00 ± 13.03	94.42 ± 14.72	96.36± 11.52	94.23 ± 12.65
$F_{MS}$		98.21 ± 03.43	96.67 ± 07.20	99.67± 01.05	100± 00	98.01 ± 03.91

**Table 6:** The effect of different activation functions on the performance of MS-MDDNet.

Activation Function	ACC%	Sen%	Spe%	AUC%	F1%
**ReLU**	**99.33 ± 01.61**	**99.00 ± 03.16**	**99.67 ± 01.05**	**99.96 ± 00.14**	**99.31 ± 01.69**
Gelu	98.67 ± 02.19	98.33 ± 03.60	99.00 ± 03.16	99.62 ± 01.05	98.65 ± 02.21
Leaky-ReLU	99.01 ± 01.79	98.00 ± 03.58	100 ± 00	99.96 ± 00.14	98.96 ± 01.87
swish	98.67 ± 03.12	97.67 ± 06.30	99.67 ± 01.05	100 ± 00	98.56 ± 03.47
Tanh	95.83 ± 08.93	96.67 ± 09.43	95.00 ± 10.57	95.90 ± 08.72	95.90 ± 09.03
clipped ReLU	99.33 ± 02.11	98.67 ± 04.22	100 ± 00	99.89 ± 00.35	99.29 ± 02.26

**Table 7:** The effect of the number of filters in the spatial analysis on the performance of MS-MDDNet.

Number of Filters (F1)	ACC%	Sen%	Spe%	AUC%	F1%
4	98.83 ± 02.49	100 ± 00	97.67 ± 04.98	98.13 ± 05.38	98.90 ± 02.35
**8**	**99.33 ± 01.61**	**99.00 ± 03.16**	**99.67 ± 01.05**	**99.96 ± 00.14**	**99.31 ± 01.69**
12	96.17 ± 07.12	100 ± 00	92.33 ± 14.23	96.74 ± 07.39	96.68 ± 06.04

**Table 8:** The effect of scales in the multiscale analysis module on MS-MDDNet’s performance.

# Scales	ACC%	Sen%	Spe%	AUC%	F1%
Without $F_{MS}$	91.24 ± 13.32	94.67 ± 12.19	87.79 ± 25.72	98.38 ± 03.89	92.07 ± 11.03
1 (125)	96.23 ± 06.66	96.33 ± 11.60	96.24 ± 08.33	98.21 ± 05.24	95.95 ± 07.58
2 (125, 62)	93.50 ± 15.12	88.33 ± 03.52	98.67 ± 04.22	99.00 ± 03.16	88.91 ± 29.22
3 (125, 62, 31)	**99.33 ± 01.61**	**99.00 ± 03.16**	**99.67 ± 01.05**	**99.96 ± 00.14**	**99.31 ± 01.69**
4 (125, 62, 31, 15)	96.06 ± 09.25	99.33 ± 02.11	92.82 ± 18.03	99.65 ± 01.12	96.68 ± 07.46

**Table 9:** The effect of the number of filters in $F_{MS}$ on MS-MDDNet’s performance.

# Filters	ACC%	Sen%	Spe%	AUC%	F1%
62, 31, 20	86.36 ± 21.16	89.00 ± 23.52	83.67 ± 35.50	84.72 ± 26.72	86.55 ± 21.41
125, 62, 31	**99.33 ± 01.61**	**99.00 ± 03.16**	**99.67 ± 01.05**	**99.96 ± 00.14**	**99.31 ± 01.69**
250, 125, 62	98.23 ± 04.15	96.33 ± 08.67	100 ± 00	100 ± 00	97.94 ± 04.96

**Table 10:** The impact of F_HFL on MS-MDDNet’s performance.

Experiment	ACC%	Sen%	Spe%	AUC%	F1%
Without $F_{HFL}$	94.33 ± 09.79	94.33 ± 16.78	94.33 ± 12.58	94.02 ± 11.34	93.74 ± 11.95
Without 1st separable	96.17 ± 08.09	100 ± 00	92.33 ± 16.18	100 ± 00	96.78 ± 06.79
Without skip-convolution	98.67 ± 04.22	100 ± 00	97.33 ± 08.43	98.48 ± 04.81	98.82 ± 03.72
With $F_{HFL}$	**99.33 ± 01.61**	**99.00 ± 03.16**	**99.67 ± 01.05**	**99.96 ± 00.14**	**99.31 ± 01.69**

**Table 11:** The effects of different pooling techniques.

Experiment	ACC%	Sen%	Spe%	AUC%	F1%
MP, GAP	95.00 ± 11.55	97.00 ± 09.49	93.00 ± 22.14	99.98 ± 7.02 × 10^−4^	95.64 ± 09.39
AP, GMP	94.86 ± 12.99	97.00 ± 06.75	92.76 ± 19.91	97.39 ± 08.26	95.57 ± 10.68
AP, GAP	**99.33 ± 01.61**	**99.00 ± 03.16**	**99.67 ± 01.05**	**99.96 ± 00.14**	**99.31 ± 01.69**

**Table 12:** The 10-fold performance of the method on the MODMA dataset.

Fold	ACC%	Sen%	Spe%	AUC%	F1%
1	100	100	100	100	100
2	95.00	90	100	99.56	94.74
3	100	100	100	100	100
4	100	100	100	100	100
5	100	100	100	100	100
6	100	100	100	100	100
7	100	100	100	100	100
8	100	100	100	100	100
9	100	100	100	100	100
10	98.33	100	96.67	100	98.36

**Table 13:** The Performance of only MS-MDDNet and Ensemble-like Classifier.

Model	ACC%	Sen%	Spe%	AUC%	F1%
Ensemble-like classifier	99.33 ± 01.61	99.00 ± 03.16	99.67 ± 01.05	99.96 ± 00.14	99.31 ± 01.69
Single model	92.00 ± 14.07	93.00 ± 22.10	91.00 ± 21.00	95.79 ± 08.20	91.09 ± 17.80

**Table 14:** Comparison of the MS-MDDNet-based method with the SOTA methods. Here #Ch stands for the number of channels.

Study/Year	#Ch	Method	Performance			
			Acc%	Spe%	AUC%	F1%
**MODMA**						
Zhang et al., 2023 [29]	64	RNN-LSTM-2D-CNN	93.77	92.18	95.32	-
Carlle et al., 2023 [39]	13	2D-CNN	50.9	-	-	-
Xu et al., 2024 [27]	128	GCN-2D-CNN	90.56	-	-	91.80
**Proposed method**	**32**	**1D-CNN**	**99.33**	**99.68**	**99.09**	**99.31**
**MUMTAZ**						
Xia et al., 2023 [28]	19	2DCNN	91.06	91.33	89.8	-
Carrle et al., 2023 [39]	13	2D-CNN	79.8	-	-	-
**Zhou et al., 2024** [33]	**20**	**2D-CNN**	**98.59**	**98.77**	**98.39**	**-**
Gan et al., 2025 [36]	19	3D-CNN	92.1	90.0	-	92.4
Wang et al., 2025 [30]	19	1D-CNN-Transformer	95.1	-	-	92.4
Proposed method	2	1D-CNN	96.21	95.69	100	97.25
**PRED + CT**						
Xu et al., 2024 [27]	-	GCN-2D-CNN	96.51	-	-	-
Wang et al., 2025 [30]	19	1D-CNN-Transformer	82.2	-	-	81.8
**Proposed method**	**6**	**1D-CNN**	**96.61**	**98.00**	**95.79**	**95.75**

**Table 15:** Testing performance of the best fold when extracting frequency bands.

Fold 7	ACC%	Sen%	Spe%	AUC%	F1%
Delta (δ)	100	100	100	100	100
Theta (θ)	52.38	0	100	100	0
Alpha (α)	52.38	0	100	100	0
Beta (β)	52.38	0	100	82.93	0
Gamma (γ)	100	100	100	100	100

**Table 16:** Testing performance of the worst fold when extracting frequency bands.

Fold 2	ACC%	Sen%	Spe%	AUC%	F1%
Delta (δ)	73.33	96.67	50.00	90.67	78.38
Theta (θ)	50.00	0	100	39.67	0
Alpha (α)	50.00	0	100	44.11	0
Beta (β)	50.00	0	100	61.22	0
Gamma (γ)	100	100	100	100	100

**Table 17:** Testing performance of the average 10 folds when extracting frequency bands.

All Folds	ACC%	Sen%	Spe%	AUC%	F1%
Delta (δ)	78.00	92.00	64.00	87.48	82.07
Theta (θ)	50.95	21.00	80.00	67.16	15.15
Alpha (α)	50.48	30.00	70.00	60.43	20.00
Beta (β)	50.48	20.00	80.00	64.01	13.33
Gamma (γ)	95.00	100	90.00	90.01	96.67

## Data Availability

The three datasets are available online at (https://modma.lzu.edu.cn/data/application/#data_1) for MODMA, (https://figshare.com/articles/dataset/EEG_Data_New/4244171) for MUMTAZ, and (https://predict.cs.unm.edu/downloads.php, d003) for PRED + CT (accessed on 22 November 2024).

## References

[1] Alzubaidi L., Zhang J., Humaidi A.J., Al-Dujaili A., Duan Y., Al-Shamma O., Santamaría J., Fadhel M.A., Al-Amidie M., Farhan L. (2021). Review of Deep Learning: Concepts, CNN Architectures, Challenges, Applications, Future Directions. J. Big Data.

[2] Sharma M., Achuth P.V., Deb D., Puthankattil S.D., Acharya U.R. (2018). An Automated Diagnosis of Depression Using Three-Channel Bandwidth-Duration Localized Wavelet Filter Bank with EEG Signals. Cogn. Syst. Res..

[3] Bashir N., Narejo S., Naz J.B.M.M., Talpur A.S. (2022). Non-Invasive EEG Based Feature Extraction Framework for Major Depressive Disorder Analysis. Int. J. Innov. Sci. Technol..

[4] Mumtaz W., Xia L., Ali S.S.A., Yasin M.A.M., Hussain M., Malik A.S. (2017). Electroencephalogram (EEG)-Based Computer-Aided Technique to Diagnose Major Depressive Disorder (MDD). Biomed. Signal Process Control.

[5] Mumtaz W., Ali S.S.A., Yasin M.A.M., Malik A.S. (2018). A Machine Learning Framework Involving EEG-Based Functional Connectivity to Diagnose Major Depressive Disorder (MDD). Med. Biol. Eng. Comput..

[6] Mumtaz W., Malik A.S. (2018). A Comparative Study of Different EEG Reference Choices for Diagnosing Unipolar Depression. Brain Topogr..

[7] Mahato S., Paul S. (2019). Detection of Major Depressive Disorder Using Linear and Non-Linear Features from EEG Signals. Microsyst. Technol..

[8] Saeedi M., Saeedi A., Maghsoudi A. (2020). Major Depressive Disorder Assessment via Enhanced K-Nearest Neighbor Method and EEG Signals. Phys. Eng. Sci. Med..

[9] Mahato S., Paul S. (2019). Classification of Depression Patients and Normal Subjects Based on Electroencephalogram (EEG) Signal Using Alpha Power and Theta Asymmetry. J. Med. Syst..

[10] Acharya U.R., Oh S.L., Hagiwara Y., Tan J.H., Adeli H., Subha D.P. (2018). Automated EEG-Based Screening of Depression Using Deep Convolutional Neural Network. Comput. Methods Programs Biomed..

[11] Mrazek V., Jawed S., Arif M., Malik A.S. (2023). Effective Eeg Feature Selection for Interpretable Mdd (Major Depressive Disorder) Classification. Proceedings of the 2023 Genetic and Evolutionary Computation Conference, GECCO 2023, Lisbon, Portugal, 15–19 July 2023.

[12] Khadidos A.O., Alyoubi K.H., Mahato S., Khadidos A.O., Mohanty S.N. (2023). Computer Aided Detection of Major Depressive Disorder (MDD) Using Electroencephalogram Signals. IEEE Access.

[13] Duan L., Duan H., Qiao Y., Sha S., Qi S., Zhang X., Huang J., Huang X., Wang C. (2020). Machine Learning Approaches for MDD Detection and Emotion Decoding Using EEG Signals. Front. Hum. Neurosci.

[14] Bachmann M., Lass J., Hinrikus H. (2017). Single Channel EEG Analysis for Detection of Depression. Biomed. Signal Process Control.

[15] Ding X., Yue X., Zheng R., Bi C., Li D., Yao G. (2019). Classifying Major Depression Patients and Healthy Controls Using EEG, Eye Tracking and Galvanic Skin Response Data. J. Affect. Disord..

[16] Aderinwale A., Tolossa G.B., Kim A.Y., Jang E.H., Lee Y., Jeon H.J., Kim H., Yu H.Y., Jeong J. (2023). Two-Channel EEG Based Diagnosis of Panic Disorder and Major Depressive Disorder Using Machine Learning and Non-Linear Dynamical Methods. Psychiatry Res. Neuroimaging.

[17] Liu X., Zhang H., Cui Y., Zhao T., Wang B., Xie X., Liang S., Sha S., Yan Y., Zhao X. (2024). EEG-Based Major Depressive Disorder Recognition by Neural Oscillation and Asymmetry. Front. Neurosci..

[18] Dong X., Yu Z., Cao W., Shi Y., Ma Q. (2020). A Survey on Ensemble Learning. Front. Comput. Sci..

[19] Murad S.A., Rahimi N. (2025). Unveiling Thoughts: A Review of Advancements in EEG Brain Signal Decoding Into Text. IEEE Trans. Cogn. Dev. Syst..

[20] Zandbagleh A., Sanei S., Azami H. (2024). Implications of Aperiodic and Periodic EEG Components in Classification of Major Depressive Disorder from Source and Electrode Perspectives. Sensors.

[21] Sun S., Li J., Chen H., Gong T., Li X., Hu B. (2020). A Study of Resting-State EEG Biomarkers for Depression Recognition. arXiv.

[22] Yang J., Zhang Z., Xiong P., Liu X. (2023). Depression Detection Based on Analysis of EEG Signals in Multi Brain Regions. J. Integr. Neurosci..

[23] Wu C.T., Huang H.C., Huang S., Chen I.M., Liao S.C., Chen C.K., Lin C., Lee S.H., Chen M.H., Tsai C.F. (2021). Resting-State EEG Signal for Major Depressive Disorder Detection: A Systematic Validation on a Large and Diverse Dataset. Biosensors.

[24] Liao S.C., Wu C.T., Huang H.C., Cheng W.T., Liu Y.H. (2017). Major Depression Detection from EEG Signals Using Kernel Eigen-Filter-Bank Common Spatial Patterns. Sensors.

[25] Bachmann M., Päeske L., Kalev K., Aarma K., Lehtmets A., Ööpik P., Lass J., Hinrikus H. (2018). Methods for Classifying Depression in Single Channel EEG Using Linear and Nonlinear Signal Analysis. Comput. Methods Programs Biomed..

[26] Sharma G., Joshi A.M., Gupta R., Cenkeramaddi L.R. (2023). DepCap: A Smart Healthcare Framework for EEG Based Depression Detection Using Time-Frequency Response and Deep Neural Network. IEEE Access.

[27] Xu C., Fan F., Shen J., Wang H., Zhang Z., Meng Q. (2024). An EEG-Based Depressive Detection Network with Adaptive Feature Learning and Channel Activation. Proceedings of the Annual Meeting of the Cognitive Science Society.

[28] Xia M., Zhang Y., Wu Y., Wang X. (2023). An End-to-End Deep Learning Model for EEG-Based Major Depressive Disorder Classification. IEEE Access.

[29] Zhang B., Wei D., Yan G., Li X., Su Y., Cai H. (2023). Spatial–Temporal EEG Fusion Based on Neural Network for Major Depressive Disorder Detection. Interdiscip. Sci..

[30] Wang Y., Zhao S., Jiang H., Li S., Li T., Pan G. (2025). M-MDD: A Multi-Task Deep Learning Framework for Major Depressive Disorder Diagnosis Using EEG. Neurocomputing.

[31] Xie Y., Yang B., Lu X., Zheng M., Fan C., Bi X., Zhou S., Li Y. (2020). Anxiety and Depression Diagnosis Method Based on Brain Networks and Convolutional Neural Networks. Proceedings of the 42nd Annual International Conference of the IEEE Engineering in Medicine & Biology Society (EMBC), Montreal, QC, Canada, 20–24 July 2020.

[32] Wang B., Fan H., Li M., Zhang F., Ji Y. (2022). Brain Wave Recognition Method for Depression in College Students Based on 2D Convolutional Neural Network. Proceedings of the ACM International Conference Proceeding Series; Association for Computing Machinery, Shanghai, China, 26–28 February 2022.

[33] Zhou Q., Sun S., Wang S., Jiang P. (2024). TanhReLU-Based Convolutional Neural Networks for MDD Classification. Front. Psychiatry.

[34] Li X., La R., Wang Y., Hu B., Zhang X. (2020). A Deep Learning Approach for Mild Depression Recognition Based on Functional Connectivity Using Electroencephalography. Front. Neurosci..

[35] Khan D.M., Yahya N., Kamel N., Faye I. (2021). Automated Diagnosis of Major Depressive Disorder Using Brain Effective Connectivity and 3D Convolutional Neural Network. IEEE Access.

[36] Gan W., Zhao R., Ma Y., Ning X. (2025). TSF-MDD: A Deep Learning Approach for Electroencephalography-Based Diagnosis of Major Depressive Disorder with Temporal–Spatial–Frequency Feature Fusion. Bioengineering.

[37] Yang C.Y., Lee H.M. (2024). Effects of the Hyperparameters on CNNs for MDD Classification Using Resting-State EEG. Electronics.

[38] Seal A., Bajpai R., Agnihotri J., Yazidi A., Herrera-Viedma E., Krejcar O. (2021). DeprNet: A Deep Convolution Neural Network Framework for Detecting Depression Using EEG. IEEE Trans. Instrum. Meas..

[39] Carrle F.P., Hollenbenders Y., Reichenbach A. (2023). Generation of Synthetic EEG Data for Training Algorithms Supporting the Diagnosis of Major Depressive Disorder. Front. Neurosci..

[40] Li X., La R., Wang Y., Niu J., Zeng S., Sun S., Zhu J. (2019). EEG-Based Mild Depression Recognition Using Convolutional Neural Network. Med. Biol. Eng. Comput..

[41] Kingma D.P., Ba J. (2017). Adam: A Method for Stochastic Optimization. arXiv.

[42] Cai H., Shuting S., Xiao H. (2022). MODMA Dataset: A Multi-Modal Open Dataset for Mental-Disorder Analysis. arXiv.

[43] Mumtaz W., Xia L., Yasin M.A.M., Ali S.S.A., Malik A.S. (2017). A Wavelet-Based Technique to Predict Treatment Outcome for Major Depressive Disorder. PLoS ONE.

[44] Cavanagh J.F., Bismark A.W., Frank M.J., Allen J.J.B. (2019). Multiple Dissociations Between Comorbid Depression and Anxiety on Reward and Punishment Processing: Evidence from Computationally Informed EEG. Comput. Psychiatry.

[45] Lashgari E., Liang D., Maoz U. (2020). Data Augmentation for Deep-Learning-Based Electroencephalography. J. Neurosci. Methods.

[46] Hosseinifard B., Moradi M.H., Rostami R. (2013). Classifying Depression Patients and Normal Subjects Using Machine Learning Techniques and Nonlinear Features from EEG Signal. Comput. Methods Programs Biomed..

[47] Mohammadi M., Al-Azab F., Raahemi B., Richards G., Jaworska N., Smith D., De La Salle S., Blier P., Knott V. (2015). Data Mining EEG Signals in Depression for Their Diagnostic Value Clinical Decision-Making, Knowledge Support Systems, and Theory. BMC Med. Inform. Decis. Mak..

[48] Tekin Erguzel T., Tas C., Cebi M. (2015). A Wrapper-Based Approach for Feature Selection and Classification of Major Depressive Disorder-Bipolar Disorders. Comput. Biol. Med..

[49] Ahmadlou M., Adeli H., Adeli A. (2012). Fractality Analysis of Frontal Brain in Major Depressive Disorder. Int. J. Psychophysiol..

[50] Li Y., Shen Y., Fan X., Huang X., Yu H., Zhao G., Ma W. (2022). A Novel EEG-Based Major Depressive Disorder Detection Framework with Two-Stage Feature Selection. BMC Med. Inform. Decis. Mak..

[51] Anik I.A., Kamal A.H.M., Kabir M.A., Uddin S., Moni M.A. (2024). A Robust Deep-Learning Model to Detect Major Depressive Disorder Utilizing EEG Signals. IEEE Trans. Artif. Intell..

[52] Fitzgerald P.J., Watson B.O. (2018). Gamma Oscillations as a Biomarker for Major Depression: An Emerging Topic. Transl. Psychiatry.

[53] Arikan M.K., Gunver M.G., Tarhan N., Metin B. (2019). High-Gamma: A Biological Marker for Suicide Attempt in Patients with Depression. J. Affect. Disord..

